# Association of Prenatal Care Expansion With Use of Antidiabetic Agents During Pregnancies Among Latina Emergency Medicaid Recipients With Gestational Diabetes

**DOI:** 10.1001/jamanetworkopen.2022.9562

**Published:** 2022-04-29

**Authors:** Maria I. Rodriguez, Ann Martinez Acevedo, Jonas J. Swartz, Aaron B. Caughey, Amy Valent, K. John McConnell

**Affiliations:** 1 Department of Obstetrics and Gynecology, Oregon Health & Science University, Portland; 2 Center for Health Systems Effectiveness, Oregon Health & Science University, Portland; 3 Department of Obstetrics and Gynecology, Duke University, Durham, North Carolina

## Abstract

**Importance:**

Access to necessary prenatal care is not guaranteed through Medicaid for some people with low income based on their immigration status. Although states have the option to extend emergency Medicaid coverage for prenatal care, many states have not expanded coverage.

**Objective:**

To evaluate whether the receipt of prenatal care services through the extension of emergency Medicaid coverage is associated with an increase in antidiabetic medication use among Latina patients with gestational diabetes.

**Design, Setting, and Participants:**

This cohort study used linked Medicaid claims and birth certificate data on live births to 4869 Latina patients from October 1, 2010, to December 31, 2019, with a difference-in-differences design to compare the rollout of prenatal care and services in Oregon in 2013 with a comparison state, South Carolina, that did not cover prenatal or postpartum care.

**Exposure:**

Medicaid coverage of prenatal care.

**Main Outcomes and Measures:**

The main outcome was the receipt of antidiabetic agents. Secondary outcomes included hypertensive disorders, cesarean delivery, postpartum contraception, and a newborn morbidity composite outcome (large size for gestational age, neonatal intensive care unit admission, and preterm birth).

**Results:**

The study sample included live births to 4869 Latina patients (mean [SD] age, 32.7 [5.5] years [range, 12-44 years]) enrolled in emergency Medicaid who were mainly aged 25 to 34 years (1499 of 2907 [51.6%]), multiparous (2626 of 2907 [90.3%]), and living in urban areas (2299 of 2907 [79.1%]). After Oregon’s policy change to offer prenatal coverage to individuals receiving emergency Medicaid, there was a large and significant increase in the receipt of antidiabetic agents among all people with diabetes during pregnancy (gestational diabetes). Prior to the policy, only 0.3% of all Latina emergency Medicaid recipients with gestational diabetes (2 of 617) received any medication (oral agents or insulin) to manage their blood glucose level. After the policy change, 28.8% of all patients with gestational diabetes (295 of 1023) received medication to manage their blood glucose level, translating to a 27.9-percentage-point increase (95% CI, 24.5-31.2 percentage points) in the receipt of antidiabetic agents in the adjusted model. The policy was also associated with a 10.4-percentage-point (95% CI, 5.3-15.5 percentage points) increase in insulin use during pregnancy among all patients with gestational diabetes. We observed an increase in postpartum contraceptive use (21.2 percentage points; 95% CI, 14.9-27.5 percentage points), the majority of which was due to postpartum sterilization (increase of 16.1 percentage points; 95% CI, 10.4-21.8 percentage points). We did not observe a significant association with gestational hypertension, cesarean births, or newborn health.

**Conclusions and Relevance:**

This retrospective cohort study suggests that expanded emergency Medicaid benefits that included prenatal care were associated with an increased use of antidiabetic medications and postpartum contraception during pregnancy.

## Key Points

**Question:**

Is there an association between prenatal care coverage in emergency Medicaid (a program of restricted Medicaid services for recent immigrants with low income who are pregnant) and the use of antidiabetic agents by pregnant Latina individuals with gestational diabetes?

**Findings:**

This cohort study of 4869 Latina patients with gestational (n = 2907) or preexisting diabetes (n = 1962) found that when Medicaid expanded coverage to include prenatal care for individuals enrolled in emergency Medicaid, receipt of antidiabetic agents and postpartum contraception significantly increased. However, there was no significant association of this prenatal care expansion with cesarean births, preterm birth, large size for gestational age, or neonatal intensive care unit admission.

**Meaning:**

This study suggests that expansion of prenatal care coverage, without postpartum care or ongoing coverage, was associated with increased use of antidiabetic agents during pregnancy.

## Teaser

This cohort study evaluates whether the receipt of prenatal care services through the extension of emergency Medicaid coverage is associated with an increase in antidiabetic medication use among people with gestational diabetes.

## Introduction

High-quality perinatal care provides evidence-based screenings and treatment to optimize maternal health from before conception to the postpartum period.^1,2,3,4,5^ It is imperative that chronic medical conditions (such as diabetes) be well under control before conception and before birth to reduce maternal and fetal risks.^6,7^ Diabetes is becoming increasingly common among individuals of reproductive age. However, many people with diabetes are unaware that they have it.^8^ The risks of inadequate prenatal care for individuals with pregestational or gestational diabetes include spontaneous abortion, fetal anomalies, macrosomia, birth injuries, cesarean delivery, neonatal intensive care unit (NICU) admission, childhood obesity, and metabolic syndrome.^6,9^

With early and regular prenatal care, diabetes can be identified and managed, which may reduce the risk for adverse outcomes.^6^ However, barriers exist to prenatal care coverage for a particular population at high risk for diabetes, immigrant women.^8,10^ Medicaid is the largest payer for obstetric care in the US and covers comprehensive prenatal and postpartum care.^11,12,13^ However, federal law requires that Medicaid recipients be citizens or permanent residents with more than 5 years of residence. Medicaid benefits are not extended to women with low income who cannot meet these citizenship requirements. Instead, these women may receive only obstetric delivery coverage through emergency Medicaid, a federal safety net program covering acute emergency events and obstetric admissions.^14^ This federal policy significantly restricts the access to prenatal and postpartum care of unauthorized immigrant women with low income.

Although federal emergency Medicaid does not cover prenatal care, states can extend coverage using state funds or the Children’s Health Insurance Program unborn child provision.^15^ Currently, 18 states, including Oregon, cover prenatal care for unauthorized immigrants, while 32 states, including South Carolina, do not.^15^

Failure to manage diabetes and optimize glycemic control during pregnancy has acute and long-term health consequences for women and children. We used 9 years of linked birth certificate and Medicaid claims data from Oregon and South Carolina to investigate the association between coverage for prenatal care and initiation of medical therapy (insulin or oral hypoglycemic agents) among Latina patients with pregestational diabetes and gestational diabetes. We also examined maternal (gestational hypertension, cesarean delivery, and postpartum contraceptive use) and neonatal (preterm birth, large size for gestational age, and NICU admission) health outcomes.

## Methods

We conducted a retrospective cohort study using linked Medicaid claims and birth certificate data from Oregon and South Carolina from October 1, 2010, to December 31, 2019. Data were obtained from both states’ Medicaid and Vital Statistics offices under a data use agreement. The study was approved by the institutional review board at Oregon Health & Science University, which waived patient consent because the data were deidentified. We followed the Strengthening the Reporting of Observational Studies in Epidemiology (STROBE) reporting guideline.^16^

Our study population was restricted to Latina patients with diabetes, enrolled in emergency Medicaid and aged 12 to 44 years who gave birth in a hospital during the study period (eFigure 1 in the Supplement). Consistent with previous studies, patients with emergency Medicaid were identified using program eligibility codes. Given that emergency Medicaid covers only specific events, no additional enrollment restrictions in the program were applied.

We included Oregon and South Carolina in our data set because both states have experienced similar increases in their immigrant population and have comparable immigrant populations in terms of size and country of origin residing in each state. During the study period, South Carolina did not cover prenatal care for emergency Medicaid recipients, and neither state provided coverage to recipients for postpartum care. Oregon rolled out prenatal care coverage for emergency Medicaid recipients on a county-by-county basis between 2008 and 2013. Our data set began in October 2010, and we excluded from analysis patients with emergency Medicaid with deliveries in Oregon counties that offered prenatal coverage prior to October 2013. This exclusion allowed us to study patients with at least 9 months of data prior to and up to 60 months after the policy change. We also excluded births with a gestational age reported of less than 23 weeks or more than 44 weeks (eFigure 1 in the Supplement).

Our primary objective was to evaluate whether expanding prenatal coverage to emergency Medicaid recipients increased the use of antidiabetic agents during pregnancy among patients with diabetes. Pregestational and gestational diabetes were identified from the birth certificate data (eTable 1 in the Supplement). We focused our primary analysis on the population with gestational diabetes because these enrollees are the group most directly affected by the policy change (Table 1).

**Table 1.** Demographic and Delivery Characteristics of Latina Emergency Medicaid Recipients With Gestational Diabetes by State (2010-2019)

Characteristic	Latina emergency Medicaid recipients, No./total No. (%)									*P* value (Oregon vs South Carolina)
	Treatment (Oregon) (n = 1834 [63.1%])			Comparison (South Carolina) (n = 1073 [36.9%])			Overall (N = 2907)			
	Before policy change	After policy change	Total	Before policy change	After policy change	Total	Before policy change	After policy change	Total	
Age, y										
<20	6/694 (0.9)	10/1140 (0.9)	16/1834 (0.9)	7/454 (1.5)	4/619 (0.6)	11/1073 (1.0)	13/1148 (1.1)	14/1759 (0.8)	27/2907 (0.9)	<.001
20-24	59/694 (8.5)	71/1140 (6.2)	130/1834 (7.1)	35/454 (7.7)	30/619 (4.8)	65/1073 (6.1)	94/1148 (8.2)	101/1759 (5.7)	195/2907 (6.7)	
25-34	365/694 (52.6)	522/1140 (45.8)	887/1834 (48.4)	287/454 (63.2)	325/619 (52.5)	612/1073 (57.0)	652/1148 (56.8)	847/1759 (48.2)	1499/2907 (51.6)	
≥35	264/694 (38.0)	537/1140 (47.1)	801/1834 (43.7)	125/454 (27.5)	260/619 (42.0)	385/1073 (35.9)	389/1148 (33.9)	797/1759 (45.3)	1186/2907 (40.8)	
Rurality										
Urban	585/694 (84.3)	967/1140 (84.8)	1552/1834 (84.6)	326/454 (71.8)	421/619 (68.0)	747/1073 (69.6)	911/1148 (79.4)	1388/1759 (78.9)	2299/2907 (79.1)	<.001
Rural	90/694 (13.0)	141/1140 (12.4)	231/1834 (12.6)	88/454 (19.4)	100/619 (16.2)	188/1073 (17.5)	178/1148 (15.5)	241/1759 (13.7)	419/2907 (14.4)	
BMI, mean (SD)	29.9 (5.8)	30.4 (5.9)	30.2 (5.8)	29.9 (6.0)	30.6 (6.5)	30.3 (6.3)	29.9 (5.9)	30.5 (6.1)	30.2 (6.0)	.69
Multiparous	601/694 (86.6)	1019/1140 (89.4)	1620/1834 (88.3)	405/454 (89.2)	601/619 (97.1)	1006/1073 (93.8)	1006/1148 (87.6)	1620/1759 (92.1)	2626/2907 (90.3)	<.001
Multifetal gestation	9/694 (1.3)	14/1140 (1.2)	23/1834 (1.3)	2/454 (0.4)	8/619 (1.3)	10/1073 (0.9)	11/1148 (1.0)	22/1759 (1.3)	33/2907 (1.1)	.54
History of previous cesarean delivery	166/694 (23.9)	295/1140 (25.9)	461/1834 (25.1)	118/454 (26.0)	183/619 (29.6)	301/1073 (28.1)	284/1148 (24.7)	478/1759 (27.2)	762/2907 (26.2)	.09
Cesarean delivery, current pregnancy	254/694 (36.6)	431/1140 (37.8)	685/1834 (37.4)	177/454 (39.0)	236/619 (38.1)	413/1073 (38.5)	431/1148 (37.5)	667/1759 (37.9)	1098/2907 (37.8)	.57
Pregnancy comorbidities										
Hypertensive disorder of pregnancy	53/694 (7.6)	97/1140 (8.5)	150/1834 (8.2)	37/454 (8.1)	68/619 (11.0)	105/1073 (9.8)	90/1148 (7.8)	165/1759 (9.4)	255/2907 (8.8)	.16
Chronic hypertension	26/694 (3.7)	44/1140 (3.9)	70/1834 (3.8)	13/454 (2.9)	29/619 (4.7)	42/1073 (3.9)	39/1148 (3.4)	73/1759 (4.2)	112/2907 (3.9)	.98

Abbreviation: BMI, body mass index (calculated as weight in kilograms divided by height in meters squared).

Antidiabetic agents (oral and injectable) were identified through National Drug Codes in the Medicaid pharmacy claims file (eTable 2 in the Supplement). We examined the use of any antidiabetic agent among pregnant patients with diabetes and the use of insulin alone, which is the first-line pharmacotherapy recommended for glycemic optimization during pregnancy. Births were identified using *International Classification of Disease, Ninth Revision* and *International Statistical Classification of Diseases and Related Health Problems, Tenth Revision* codes as well as *Current Procedural Terminology* codes and using a previously published algorithm.^17,18,19^

Our secondary health outcomes included measures of maternal and newborn health. We examined the association of prenatal care expansion with gestational hypertension, cesarean birth, and postpartum contraception. Because the policy change is specific to the prenatal and intrapartum periods, we examined rates of postpartum sterilization separately from overall contraceptive use. These outcomes were captured from Medicaid claims data (eTable 2 in the Supplement) and corroborated by birth certificate data. We captured newborn health outcomes from the birth certificate data and categorized preterm birth, NICU admission, and large size for gestational age as a composite measure.

We abstracted demographic and clinical information from the birth certificate files and claims data. Demographic data obtained from claims records included maternal age, zip code of residence, and immigration status. Zip codes were classified as rural using Rural-Urban Commuting Area Codes.^20^ Emergency Medicaid is a proxy for immigration status; enrollment in the program is contingent on being a noncitizen.^14^ Previous research has demonstrated that the population receiving emergency Medicaid is predominantly Latina women, and there are well-documented racial and ethnic and economic disparities in maternal care and outcomes.^17,21^ We therefore focused our outcome on Latina patients to mitigate confounding from the described race and ethnicity differences in the incidence and severity of diabetes during pregnancy. We also captured information on maternal age, multiparity, and body mass index.^22^

### Statistical Analysis

We estimated the outcome of Oregon’s prenatal coverage policy using a difference-in-differences analysis. We isolated 21 counties in Oregon that implemented the policy in October 2013 to compare with South Carolina. We adjusted for maternal age and body mass index in models for all of our outcomes, and we adjusted for nulliparity in our models for gestational hypertension and cesarean delivery. Standard errors were clustered at the county level.^23,24,25^ We conducted 2-sided tests with an α level of .05.^26^

Our tests for nonparallel trends did not indicate significant differences in outcomes prior to the policy change (Table 2 and Table 3; eTable 3 in the Supplement). We included a missing category for observations missing data on maternal county of residence in vital statistics records. Individuals receiving emergency Medicaid may have fragmented Medicaid coverage, which may affect the ability to both identify and correctly classify diabetes as pregestational or gestational. We conducted a sensitivity analysis in which we examined all study outcomes among patients with any type of diabetes (gestational and pregestational). Results were consistent with the main models and are reported in eTable 3 and eFigures 2 to 7 in the Supplement. Our analyses were conducted using R, version 4.0.3 (R Group for Statistical Computing).

**Table 2.** Medication Initiation Among Latina Emergency Medicaid Recipients With Gestational Diabetes After Prenatal Care Expansion (2010-2019)

Medication initiation	Treatment (Oregon) (n = 1640)			Comparison (South Carolina) (n = 930)			Adjusted difference-in-differences estimate, percentage points (95% CI)
	Participants before policy change (n = 617), No. (%)	Participants after policy change (n = 1023), No. (%)	Difference, %	Participants before policy change (n = 387), No. (%)	Participants after policy change (n = 543), No. (%)	Difference, %	
Any antidiabetic medication^a^^,^^b^^,^^c^	2 (0.3)	295 (28.8)	28.5	2 (0.5)	4 (0.7)	0.2	27.9 (24.5-31.2)
Insulin^a^^,^^b^^,^^d^	0	1106 (0.4)	10.4	2 (0.5)	0	−0.5	10.4 (5.3-15.5)

^a^ At any point during pregnancy.

^b^ Estimates adjusted for maternal age and body mass index.

^c^ Pretest of parallel trends, *P* = .88.

^d^ Pretest of parallel trends, *P* = .99.

**Table 3.** Maternal and Neonatal Health Outcomes Among Latina Emergency Medicaid Recipients After Expansion of Prenatal Care (2010-2019)

Outcome	Treatment (Oregon) (n = 1640)			Comparison (South Carolina) (n = 930)			Adjusted difference-in-differences estimate, percentage points (95% CI)
	Participants before policy change (n = 617), No. (%)	Participants after policy change (n = 1023), No. (%)	Difference, %	Participants before policy change (n = 387), No. (%)	Participants after policy change (n = 543), No. (%)	Difference, %	
Gestational hypertension^a^^,^^b^^,^^c^^,^^d^	45 (7.3)	81 (7.9)	0.6	29 (7.5)	57 (10.5)	3.0	−2.8 (−7.1 to 1.5)
Cesarean birth^a^^,^^b^^,^^c^^,^^e^	216 (35.0)	379 (37.0)	2.0	150 (38.8)	205 (37.8)	−1.0	3.8 (−4.8 to 12.4)
Postpartum contraception (all)^b^^,^^f^	0	235 (23.0)	23.0	14 (3.6)	23 (4.2)	0.6	21.2 (14.9 to 27.5)
Postpartum sterilization^b^^,^^g^	0	154 (15.1)	15.1	11 (2.8)	9 (1.7)	−1.1	16.1 (10.4 to 21.8)
Infant composite morbidity^b^^,^^h^	176 (28.5)	186 (26.9)	−1.6	117 (30.2)	186 (34.3)	4.1	−4.1 (−12.7 to 4.5)

^a^ At any point during pregnancy.

^b^ Estimates adjusted for maternal age and body mass index.

^c^ Estimates adjusted for nulliparity.

^d^ Pretest of parallel trends, *P* = .51.

^e^ Pretest of parallel trends, *P* = .26.

^f^ Pretest of parallel trends, *P* = .80.

^g^ Pretest of parallel trends, *P* = .44.

^h^ Pretest of parallel trends, *P* = .54.

## Results

Our study sample included live births to 4869 Latina patients (mean [SD] age, 32.7 [5.5] years) enrolled in emergency Medicaid who were mainly aged 25 to 34 years (1499 of 2907 [51.6%]), multiparous (2626 of 2907 [90.3%]), and living in urban areas (2299 of 2907 [79.1%]) (Table 1). More births in our sample occurred in the Oregon cohort group than in the South Carolina comparison group (1834 of 2907 [63.1%] vs 1073 of 2907 [36.9%]). In both groups, most births were to multiparous patients, were delivered vaginally, and were at term. Patients giving birth in Oregon were more likely than those giving birth in South Carolina to be aged 35 years or older (801 of 1834 [43.7%] vs 385 of 1073 [35.9%]; *P* < .001) and living in urban counties (1552 of 1834 [84.6%] vs 747 of 1073 [69.6%]; *P* < .001).

**Figure 1.**
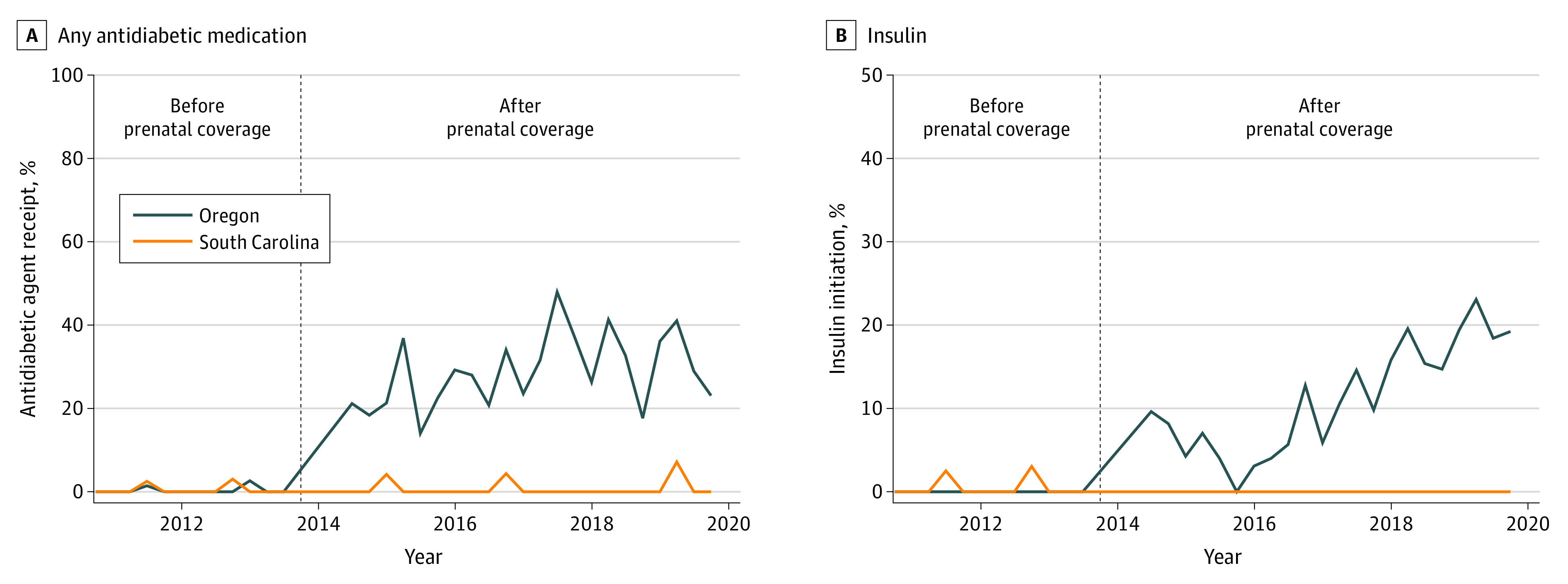
Difference-in-Differences Estimates of Any Medication Initiation and Insulin Initiation Among Latina Emergency Medicaid Recipients With Gestational Diabetes, 2010-2019

Among patients in Oregon with gestational diabetes who were covered by emergency Medicaid, only 0.3% (2 of 617) used any antidiabetic agent during pregnancy prior to the policy (Table 2). After the policy change, this percentage increased to 28.8% (295 of 1023). In our adjusted difference-in-differences model, prenatal care coverage was associated with a 27.9-percentage-point increase in antidiabetic medication use (95% CI, 24.5-31.2 percentage points) (Figure 1A). Prenatal care coverage was associated with a 10.4-percentage-point increase in insulin use (95% CI, 5.3-15.5 percentage points) (Figure 1B) among individuals with gestational diabetes.

**Figure 2.**
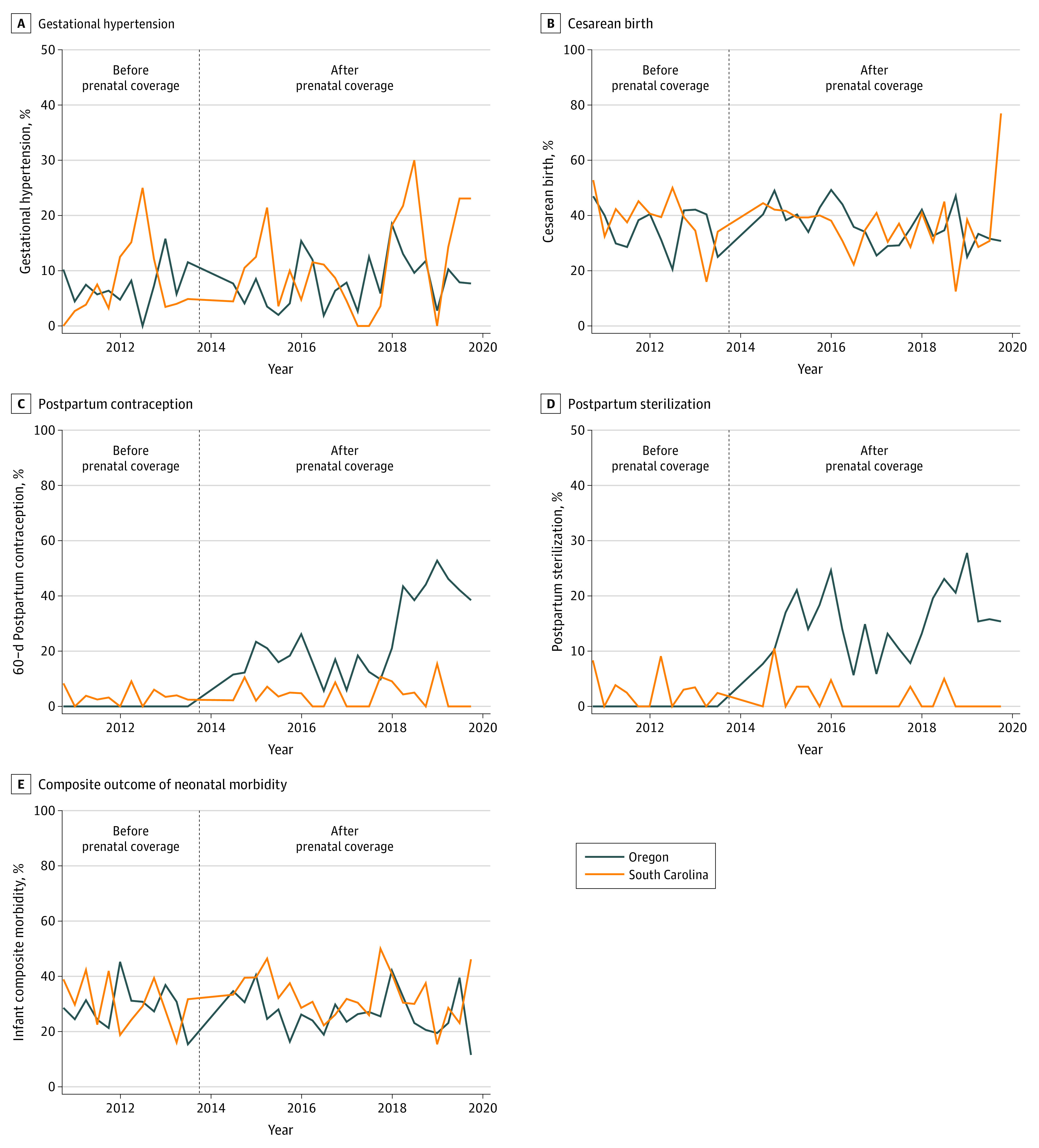
Difference-in-Differences Estimates of Maternal and Neonatal Health Outcomes After Prenatal Care Expansion Among Latina Emergency Medicaid Enrollees With Gestational Diabetes, 2010-2019 The composite outcome of neonatal morbidity includes neonatal intensive care unit admission, preterm birth, and large for gestational age.

We then examined additional maternal health outcomes (Table 3). Prenatal care coverage was not associated with a significant change in gestational hypertension (adjusted difference-in-differences model, –2.8 percentage points [95% CI, –7.1 to 1.5 percentage points]) or cesarean birth (adjusted difference-in-differences model, 3.8 percentage points [95% CI, –4.8 to 12.4 percentage points]) during pregnancy (Figure 2A and B). We observed a marked increase in the use of postpartum contraception among patients with gestational diabetes when prenatal care was covered (adjusted difference-in-differences model, 21.2 percentage points [95% CI, 14.9-27.5 percentage points]) (Table 3; Figure 2C). The increase in postpartum contraception was largely associated with an increase in postpartum sterilization (Figure 2D). Prior to the policy change, none of the patients with emergency Medicaid in Oregon received postpartum sterilization. Rates of postpartum sterilization increased to 15.1% (154 of 1023) after the policy change. In our adjusted difference-in-differences model, this change corresponded to an increase of 16.1 percentage points in postpartum sterilization (95% CI, 10.4-21.8 percentage points) (Table 3). We examined newborn health outcomes as a composite measure of preterm birth, large size for gestational age, and NICU admission (Figure 2D). We did not observe a significant reduction in neonatal morbidity after the policy change (adjusted difference-in-differences model, −4.1 percentage points; 95% CI, −12.7 to 4.5 percentage points) (Table 3).

## Discussion

The inclusion of prenatal coverage within emergency Medicaid was associated with a rapid and substantial increase in the receipt of antidiabetic agents for individuals with pregnancies complicated by diabetes. Diagnosis and treatment of pregestational diabetes and gestational diabetes may reduce maternal and neonatal morbidity in the short and long term.^6,9,27^ Our findings suggest that restricting access to prenatal care for individuals with low income based on citizenship status presents barriers to accessing important preventive health care during pregnancy.

Pregnant women with low income who are unauthorized immigrants face barriers to access to health care based on their state of residence.^15^ Diabetes affects an increasing proportion of women during pregnancy and confers risks for both those women and their offspring. Underdiagnosis may be associated with worse outcomes.^28,29,30,31^ Comprehensive, high-quality care is paramount for both pregestational and gestational diabetes during pregnancy. Both lifestyle interventions and pharmacologic agents to improve glycemia reduces the risk for adverse perinatal outcomes and may reduce long-term health outcomes for women and their offspring.^28,29,32^

Medicaid policy is a structural determinant of perinatal and reproductive health and finances almost half of births in the US.^33^ Medicaid expansion under the Patient Protection and Affordable Care Act significantly reduced uninsurance rates, improved adequacy of prenatal care, and reduced parental stress.^34,35,36^ Nationally, supporters have advocated for 12 months of continuous postpartum Medicaid coverage, recognizing the critical role this period plays for both maternal and infant health.^37^ Poorly controlled diabetes and hypertension are risk factors for the most common causes of severe maternal morbidity and mortality in the US. These risks are exacerbated among the underinsured and uninsured populations.^38,39^ The Centers for Disease Control and Prevention estimates that 60% of severe maternal morbidity and mortality combined in the US is preventable and identifies timely access to high-quality perinatal care as critical to preventing maternal death and disability.^40^

However, immigrant women with low income remain largely excluded from Medicaid coverage, which is a missed opportunity to avert preventable maternal morbidity and mortality and reduce long-term chronic health conditions.^17,41^ Nearly 20% of pregnant women enrolled in emergency Medicaid have diabetes or hypertension.^17^ We found that expanded coverage to include prenatal care and services significantly increased the use of antidiabetic agents among patients with diabetes during pregnancy.

Short interpregnancy intervals (<18 months) have been associated with a range of poor maternal health outcomes, in particular, unintended pregnancy, increased risk of obesity, and progression to type 2 diabetes.^17,42,43^ Most (74.4%) short-interval pregnancies are mistimed or unwanted.^43^ Contraception is a highly effective strategy to allow individuals to determine if and when they wish to become pregnant; however, barriers exist to accessing a full range of methods for the underinsured and uninsured individuals.^44^ Prior to passage of prenatal care coverage, only 1.4% of emergency Medicaid recipients with diabetes (14 of 1004) received a mostly effective or moderately effective form of contraception. After the policy change, we observed an increase of 21.2 percentage points (95% CI, 14.9-27.5 percentage points) in contraceptive use among patients with pregnancies complicated by diabetes. For individuals with diabetes, interpregnancy care is critical to optimize maternal and child health. Approximately 70% of individuals with a diagnosis of gestational diabetes will develop type 2 diabetes in their lifetime.^28,45,46,47,48,49,50^ Ensuring that individuals have the choice of their method of contraception may help reduce progression to type 2 diabetes and may assist in optimizing glycemic control prior to the subsequent pregnancy.

Nearly three-fourths of the increase in contraceptive use observed was associated with an increase in postpartum tubal sterilization. The postpolicy rate of tubal sterilization observed in Oregon was 15.1%, which is nearly twice the rate reported in the general postpartum population in the US (8%-9%).^51^ Although our finding may reflect an increased preference for permanent contraception among a medically complex population, it is also possible that the higher rate of postpartum sterilization observed is associated with the lack of access to reversible methods. Policy options to expand prenatal care do not include comprehensive postpartum coverage. The immigrant community in the US has experienced reproductive coercion and forced sterilization; it is critical from a human rights and public health perspective that all individuals have a free and informed choice to use contraception.^52,53^

### Limitations

Our study should be interpreted with the following limitations in mind. Administrative claims data may be subject to errors in coding. However, our study is strengthened by the use of 2 distinct data sources, Medicaid claims and birth certificate data, which allowed us to corroborate health outcomes and improve the demographic information available. We used data from 2 states, Oregon and South Carolina, which may limit our generalizability to other areas. We were not able to capture subsequent births to patients who moved out of state or switched to a private payer or who may have received care through charity programs, safety-net clinics, or federally qualified health centers. Our study is subject to ascertainment bias; patients with ongoing insurance coverage are more likely to receive a diagnosis of pregestational diabetes owing to increased exposure to the health care system. Our population of interest, emergency Medicaid recipients, was less likely to receive a diagnosis of any type of diabetes overall and more likely to receive a diagnosis of gestational diabetes after the policy change. We addressed this limitation by conducting a sensitivity analysis in which we examined outcomes for births complicated by gestational diabetes or pregestational diabetes together. Results were consistent with our main model. We suspect, but cannot know for certain, that claims covered prior to the policy change were covered as part of a presumptive eligibility pathway. We were underpowered to examine outcomes for patients with pregestational diabetes separately or to measure many neonatal health outcomes.

## Conclusions

Immigrant women experience several layers of barriers to accessing health care; restricted access to health care during pregnancy has multigenerational consequences.^54^ Our study highlights the benefits associated with covering prenatal and intrapartum care for women with pregnancies complicated by diabetes. We found that expanded coverage for prenatal care was associated with a significant increase in the use of antidiabetic medications during pregnancy, decreased incidence of gestational hypertensive disorders, and increased use of postpartum contraception. State and federal Medicaid programs should cover pregnancy-related care for all individuals with demonstrated financial need.

## Notes

Supplement. **eTable 1.** Demographics and Delivery Characteristics of Emergency Medicaid Births by State (2010-2019) **eTable 2.** National Drug Codes for Antidiabetic Agents **eTable 3.** Study Outcomes Among Emergency Medicaid Latina Recipients With Preexisting or Gestational Diabetes Following Prenatal Care Expansion from 2010-2019 **eFigure 1.** Cohort Creation **eFigure 2.** Adjusted Trend Estimates of any Medication Initiation During Prenatal Period Among Emergency Medicaid Recipients With Pregestational or Gestational Diabetes (2010-2019) **eFigure 3.** Adjusted Trend Estimates of Insulin Initiation During Prenatal Period Among Emergency Medicaid Recipients With Pregestational or Gestational Diabetes (2010-2019) **eFigure 4.** Adjusted Trend Estimates of Gestational Hypertension During Prenatal Period Among Emergency Medicaid Recipients With Pregestational or Gestational Diabetes (2010-2019) **eFigure 5.** Adjusted Trend Estimates of Cesarean Birth During Prenatal Period Among Emergency Medicaid Recipients With Pregestational or Gestational Diabetes (2010-2019) **eFigure 6.** Adjusted Trend Estimates of Postpartum Contraception During Prenatal Period Among Emergency Medicaid Recipients With Pregestational or Gestational Diabetes (2010-2019) **eFigure 7.** Adjusted Trend Estimates of Postpartum Sterilization During Prenatal Period Among Emergency Medicaid Recipients With Pregestational or Gestational Diabetes (2010-2019) **eFigure 8.** Adjusted Trend Estimates of Infant Morbidity During Prenatal Period Among Emergency Medicaid Recipients With Pregestational or Gestational Diabetes (2010-2019)
