# Supplementary material for: Association of Prenatal Care Expansion With Use of Antidiabetic Agents During Pregnancies Among Latina Emergency Medicaid Recipients With Gestational Diabetes
Source: JAMA Netw Open. 2022 Apr 29;5(4):e229562. doi: 10.1001/jamanetworkopen.2022.9562 (PMC9055460; doi:10.1001/jamanetworkopen.2022.9562)
Supplement: Supplement. — eTable 1. Demographics and Delivery Characteristics of Emergency Medicaid Births by State (2010-2019) eTable 2. National Drug Codes for Antidiabetic Agents eTable 3. Study Outcomes Among Emergency Medicaid Latina Recipients With Preexisting or Gestational Diabetes Following Prenatal Care Expansion from 2010-2019 eFigure 1. Cohort Creation eFigure 2. Adjusted Trend Estimates of any Medication Initiation During Prenatal Period Among Emergency Medicaid Recipients With Pregestational or Gestational Diabetes (2010-2019) eFigure 3. Adjusted Trend Estimates of Insulin Initiation During Prenatal Period Among Emergency Medicaid Recipients With Pregestational or Gestational Diabetes (2010-2019) eFigure 4. Adjusted Trend Estimates of Gestational Hypertension During Prenatal Period Among Emergency Medicaid Recipients With Pregestational or Gestational Diabetes (2010-2019) eFigure 5. Adjusted Trend Estimates of Cesarean Birth During Prenatal Period Among Emergency Medicaid Recipients With Pregestational or Gestational Diabetes (2010-2019) eFigure 6. Adjusted Trend Estimates of Postpartum Contraception During Prenatal Period Among Emergency Medicaid Recipients With Pregestational or Gestational Diabetes (2010-2019) eFigure 7. Adjusted Trend Estimates of Postpartum Sterilization During Prenatal Period Among Emergency Medicaid Recipients With Pregestational or Gestational Diabetes (2010-2019) eFigure 8. Adjusted Trend Estimates of Infant Morbidity During Prenatal Period Among Emergency Medicaid Recipients With Pregestational or Gestational Diabetes (2010-2019) [file jamanetwopen-e229562-s001.pdf]

Supplementary Online Content

Rodriguez MI, Martinez Acevedo A, Swartz JJ, Caughey AB, Valent A, McConnell KJ. Association of prenatal care expansion with use of antidiabetic agents during pregnancies among Latina emergency Medicaid recipients with gestational diabetes. *JAMA Netw Open.* 2022;5(4):e229562. doi:10.1001/jamanetworkopen.2022.9562

eTable 1. Demographics and Delivery Characteristics of Emergency Medicaid Births by State (2010-2019)
eTable 2. National Drug Codes for Antidiabetic Agents
eTable 3. Study Outcomes Among Emergency Medicaid Latina Recipients With Preexisting or Gestational Diabetes Following Prenatal Care Expansion from 2010-2019
eFigure 1. Cohort Creation
eFigure 2. Adjusted Trend Estimates of any Medication Initiation During Prenatal Period Among Emergency Medicaid Recipients With Pregestational or Gestational Diabetes (2010-2019)
eFigure 3. Adjusted Trend Estimates of Insulin Initiation During Prenatal Period Among Emergency Medicaid Recipients With Pregestational or Gestational Diabetes (2010-2019)
eFigure 4. Adjusted Trend Estimates of Gestational Hypertension During Prenatal Period Among Emergency Medicaid Recipients With Pregestational or Gestational Diabetes (2010-2019)
eFigure 5. Adjusted Trend Estimates of Cesarean Birth During Prenatal Period Among Emergency Medicaid Recipients With Pregestational or Gestational Diabetes (2010-2019)
eFigure 6. Adjusted Trend Estimates of Postpartum Contraception During Prenatal Period Among Emergency Medicaid Recipients With Pregestational or Gestational Diabetes (2010-2019)
eFigure 7. Adjusted Trend Estimates of Postpartum Sterilization During Prenatal Period Among Emergency Medicaid Recipients With Pregestational or Gestational Diabetes (2010-2019)
eFigure 8. Adjusted Trend Estimates of Infant Morbidity During Prenatal Period Among Emergency Medicaid Recipients With Pregestational or Gestational Diabetes (2010-2019)

This supplementary material has been provided by the authors to give readers additional information about their work.

© 2022 Rodriguez MI et al. *JAMA Network Open.*

eTable 1. Demographics and Delivery Characteristics of Emergency Medicaid Births by State (2010-2019)

**Maternal Characteristics**	**Treatment (Oregon)**	**Comparison (South Carolina)**	**Overall**	***P* value**
	**(N=26 459 [57%])**	**(N=20 096 [43%])**	**(N=46 555)**	
	No. (%)	No. (%)	**No. (%)**	
**Age**				<0.001
<20	1 084 (4.1)	1 232 (6.1)	2 316 (5.0)	
20-24	5 189 (19.6)	4 159 (20.7)	9 348 (20.1)	
25-34	14 358 (54.3)	11 368 (56.6)	25 726 (55.3)	
35 & up	5 828 (21.9)	3 337 (16.6)	9 165 (19.7)	
**Race**				<0.001
Hispanic	22 626 (85.5)	11 955 (59.5)	34 581 (74.3)	
Non-Hispanic Black	401 (1.5)	285 (1.4)	686 (1.5)	
Non-Hispanic White	1 025 (3.9)	544 (2.7)	1 569 (3.4)	
Other/Unknown*	2 407 (9.1)	7 312 (36.4)	9 719 (20.9)	
**Rurality**				<0.001
Urban	22 016 (83.2)	12 928 (64.3)	34 944 (75.1)	
Rural	3 929 (14.8)	4 223 (21.0)	8 152 (17.5)	
Missing	514 (1.9)	2 945 (14.7)	3 459 (7.4)	
**Body Mass Index (mean (SD))**	27.3 (5.48)	27.1 (5.34)	27.2 (5.42)	<0.001
**Multiparous**	20 789 (78.6)	15 244 (75.9)	36 033 (77.4)	<0.001
**Multifetal gestation**	267 (1.0)	181 (0.9)	448 (1.0)	0.255
**History of previous cesarean**	5 110 (19.3)	3 536 (17.6)	8 646 (18.6)	<0.001
**Cesarean delivery, current pregnancy**	7 298 (27.6)	5 522 (27.5)	12 820 (27.5)	0.811

© 2022 Rodriguez MI et al. *JAMA Network Open.*

**Pregnancy comorbidities**				
Hypertensive disorder of pregnancy	1 251 (4.7)	919 (4.6)	2 170 (4.7)	0.445
Chronic hypertension	350 (1.3)	198 (1.0)	548 (1.2)	0.001
Gestational Diabetes	3 695 (14.0)	1 598 (8.0)	5 293 (11.4)	<0.001
Pre-existing diabetes	467 (1.8)	237 (1.2)	704 (1.5)	<0.001

© 2022 Rodriguez MI et al. *JAMA Network Open.*

eTable 2. National Drug Codes for Antidiabetic Agents

**Outcome Measure**	**Coding System**	**Codes**
Insulin		"00169330312" "00169633910" "00169750111" "32849050081" "32849050087" "54868277700" "54868605400" "68258889903" "00169368213" "00169368512" "00169369619" "54868520100" "54868532700" "54868532701" "00169255013" "00169255097" "00169266015" "00169266097" "00169368712" "00169643810" "00169643890" "00169643897" "00169643910" "54569630000" "54569630100" "54868011200" "54868588300" "00024586903" "00088221905" "00088222033" "00088222052" "00088222060" "49999099410" "54569560500" "54569646200" "54569662500" "54868462600" "54868576500" "54868623100" "68115083910" "00088250033" "00088250052" "00088250205" "00002831001" "54569165101" "54868142801" "32849075601" "00002831101" "00169244710" "32849080601" "54569281700" "00002751001" "00002751017" "00002751501" "00002751559" "00002751601" "00002751659" "00002771201" "00002771227" "00002771299" "00002872501" "00002872559" "00002879901" "00002879959" "35356010200" "54868510800" "54868583600" "68115074610" "00002751101" "00002751201" "00002879301" "00002879359" "00002879401" "00002879459" "00002879701" "00002879759" "00002879801" "00002879857" "00002879859" "54569532100" "54868438100" "00002871501" "00002871517" "00002871591" "00002871759" "00002877001" "00002877059" "00002880301" "00002880359" "00002951501" "00169001771" "00169183702" "00169183711" "00169183717" "00169183718" "00169231721" "00169347718" "32849070801" "32849070802" "32849070803" "32849070807" "49999099310" "54569291800" "54569291801" "54569346700" "54569346701" "54868274600" "54868347400" "54868582400" "55045350801" "59060023174" "59060183702" "59060231704" "00002831501"

© 2022 Rodriguez MI et al. *JAMA Network Open.*

		"00002831517" "00002831591" "00002831759" "00002873001" "00002873059" "00002880501"<br><br>"00002880559" "00169004571" "00169183402" "00169183411" "00169183417" "00169183418" "00169231421" "00169347418" "32849070601" "32849070602" "32849070607" "54569231800" "54569231801" "54569383500" "54569383501" "54868142901" "54868238001" "58016478801" "59060023144" "59060183402" "59060231404" "68115072905" "00169011101" "32849071701" "00002821501" "00002821517" "00002821591" "00002821759" "00002850101" "00002882401" "00002882427" "00024587490" "00024588236" "00024588463" "00024589463" "00169004471" "00169183302" "00169183311" "00169183317" "00169183318" "00169231321" "00169347318" "00403344918" "32849070701" "32849070702" "32849070707" "47918087490" "47918088018" "47918088236" "47918088463" "47918089463" "54569231900" "54569231901" "54569383300" "54569383301" "54868359800" "54868361900" "55045350601" "59060183302" "68115070905"<br><br>"68115072810" "00002821001" "54569295100" "54569295101" "54868208901" "00169007011" "32849072701" "00069005019" "00069005053" "00069070737" "00069072437" "32849075501" "00002861501" "00002841501" "00169183511" "32849070501" "54569383400" "54569383401" "00002841001" "00002841101" "00169244210" "32849080501" "32849075701" "00002821101" "00169244010" "32849080701" "00002850001" "00069005085"
Oral anti-diabetic medications		"00087606005" "00087606010" "00087606313" "00087606314" "00087606413" "00087607005" "00087607010" "00087607111" "00087607112" "00093104801" "00093104805" "00093104810""00093104819" "00093104893" "00093104898" "00093104901" "00093104905" "00093104910" "00093104919" "00093104993" "00093104998" "00093721201" "00093721401"

		"00093721405" "00093721410" "00093721498" "00093726701" "00093726710" "00172433000" "00172433010" "00172433060" "00172433070" "00172433080" "00172433100" "00172433110" "00172433160" "00172433170" "00172433180" "00172433188" "00172443210" "00172443260" "00172443270" "00172443280" "00172443500" "00172443510" "00172443560" "00172443570" "00185021301""00185021305" "00185021501" "00185021505" "00185022101" "00185022105" "00185441601" "00185441605" "00228265711" "00228265750" "00228271511" "00228271550" "00228271811" "00228271850" "00228272811" "00228274011" "00247144330" "00378023401" "00378023405" "00378024001" "00378024401" "00378035001" "00378035201" "00378035205" "00378600191" "00378600291" "00378718505" "00378718605" "00378718705" "00406202801" "00406202805" "00406202810" "00406202901" "00406202905" "00406202910" "00406203001" "00406203005" "00406203010" "00440573900" "00440573901" "00440573905" "00440573981" "00440573985" "00440573990" "00440573992" "00440574500" "00440574501" "00440574505" "00440574581" "00440574585" "00440574590" "00440574592" "00440574600" "00440574601" "00440574605" "00440574681" "00440574685" "00440574690" "00440574692" "00440756295" "00440773914" "00440773960" "00440773990" "00440773992" "00440773994" "00440773995" "00440774590" "00440774592" "00440774690" "00440774692" "00440774890" "00440774892" "00440774899""00555010702" "00555038502" "00555038504" "00555038602" "00555038702" "00591245501" "00591245505" "00591271301" "00591271305" "00591271960" "00591272060" "00591277501" "00591277525" "00603446721" "00603446728" "00603446732" "00603446821" "00603446828" "00603446832" "00603446921" "00603446928" "00603446932" "00615458053" "00615458063" "00781505001" "00781505005" "00781505010" "00781505061" "00781505101" "00781505105" "00781505161" "00781505201"

© 2022 Rodriguez MI et al. *JAMA Network Open.*

		"00781505205" "00781505261" "00781505501" "00904560118" "00904560152" "00904560154" "00904560161" "00904560180" "00904560189" "00904560193" "00904560240" "00904560253" "00904560261" "00904560289" "00904560293" "00904560340" "00904560352" "00904560361" "00904560389" "00904560393" "00904563461" "00904563561" "00904563661" "00904579461" "00904579561" "00904584914" "00904584918" "00904584940""00904584952" "00904584953" "00904584954" "00904584980" "00904584989" "00904584993" "00904585040" "00904585052" "00904585053" "00904585089" "00904585093" "00904585140" "00904585152" "00904585189" "00904585193" "00904609061" "00904609161" "00904609261" "00904610740" "00904610761" "00904610860" "00904610861" "00904632661" "00904632761" "00904632861" "00904634314" "00904634318" "00904634325" "00904634340" "00904634352" "00904634353" "00904634354" "00904634360" "00904634380" "00904634389" "00904634393" "00904634440" "00904634452" "00904634453" "00904634489" "00904634493" "00904634540" "00904634552" "00904634589" "00904634593" "10544024802" "10544024830" "10544024860" "10544024890" "10544052130" "10544058960" "10544063030" "10544063090" "10544094430" "10544094490" "10544094790" "10631020601" "10631020602" "10631023801" "10631023802""13913000213" "13913000316" "14565020210" "14565020250" "15338040060" "16590031330" "16590031360" "16590031372" "16590031382" "16590031390" "17236013201" "17236013205" "17236013301" "20091053101" "20091053105" "20091053110" "20091053301" "20091053305" "20091053310" "20091053501" "20091053505" "20091053510" "21695047100" "21695047130" "21695047160" "21695047172" "21695047178" "21695047190" "21695047230" "21695047260" "21695047300" "21695047330" "21695047360" "21695047378" "21695047390" "21695082830" "21695082860" "21695082890" "23155010201" "23155010205" "23155010206"

© 2022 Rodriguez MI et al. *JAMA Network Open.*

		"23155010210" "23155010301" "23155010305" "23155010306" "23155010310" "23155010401" "23155010405" "23155010406" "23155010410" "23490683801" "23490683802" "23490683803" "23490683804" "23490683901" "23490683902" "23490726001" "23490726002" "23490726003" "23490726004""23490745806" "23629015110" "24658029005" "24658029012" "24658029018" "24658029027" "24658029036" "24658029046" "24658029060" "24658029090" "24658029205" "24658029218" "24658029260" "24658029290" "29033001801" "29033001805" "29033001810" "29033002101" "33358023400" "33358023430" "33358023460" "33358023560" "33358023630" "33358023660" "33358023730" "33358023760" "35356026928" "35356069160" "35356079230" "35356088630" "35356088660" "35356088690" "35356092290" "35356095930" "35356095960" "35356095990" "42291059218" "42291059260" "42291059318" "42291059360" "42291060510" "42291060512" "42291060518" "42291060527" "42291060536" "42291060545" "42291060560" "42291060590" "42291060610" "42291060618" "42291060627" "42291060690" "42291060710" "42291060718" "42291060760" "42291060790" "42291061010" "42291061018" "42291061036" "42291061090""42291061118" "42291061127" "42291061150" "42291061190" "42806021305" "42806021310" "42806021501" "42806021505" "42806022105" "43063001201" "43063001260" "43063001286" "43063001290" "43063001293" "43063001294" "43063037230" "43063042830" "43063042890" "43063042893" "43063042894" "43063042993" "43063042994" "43063043030" "43063043060" "43063043090" "43063043093" "43063043094" "43063043098" "43063052720" "43063052730" "43063052760" "43063052790" "43063052793" "43063052794" "43063053986" "43063053990" "43063053993" "43063053994" "43353006553" "43353006560" "43353006580" "43353006594" "43353008260" "43353008280" "43353008292" "43353009880" "43353011480" "43353012830" "43353012845" "43353012853"

© 2022 Rodriguez MI et al. *JAMA Network Open.*

		"43353012860" "43353012873" "43353012880" "43353012886" "43353017160" "43353034030" "43353034053" "43353034060" "43353034070" "43353034075""43353034080" "43353034092" "43353034094" "43353034096" "43353034453" "43353034460" "43353034480" "43353034492" "43353034930" "43353034945" "43353034953" "43353034960" "43353034973" "43353034980" "43353034986" "43353047730" "43353047753" "43353047760" "43353047770" "43353047780" "43353047792" "43353047794" "43353047796" "43353051430" "43353051445" "43353051453" "43353051460" "43353051473" "43353051480" "43353051486" "43353052060" "43353052080" "43353053060" "43353053080" "43353058594" "43353058930" "43353058953" "43353058960" "43353058970" "43353058975" "43353058980" "43353058992" "43353058994" "43353058996" "43353081153" "43353081160" "43353081180" "43353081192" "43353084230" "43353084253" "43353084260" "43353084280" "43353088545" "43353088553" "43353088560" "43353088573" "43353088580" "43353088586" "43353089430" "43353093630""43353093645" "43353093673" "43353093686" "43353098230" "43353098253" "43353098260" "43353098280" "43353098292" "43353098294" "43353098296" "43353098730" "43353098745" "43353098753" "43353098760" "43353098773" "43353098780" "43353098786" "43547024810" "43547024811" "43547024850" "43547024910" "43547024911" "43547024950" "43547025010" "43547025011" "43547025050" "43547032010" "43547032011" "43547032050" "43547032110" "43547032150" "43547032210" "43547032250" "43547035710" "43547035711" "43547035750" "43547035810" "43547035850" "43547035910" "43547035950" "49483062301" "49483062350" "49483062401" "49884073601" "49884073605" "49884073701" "49884073705" "49884073801" "49884073805" "49884073901" "49884073905" "49884074001" "49884074005" "49884074101" "49884074105" "49884092101" "49884092105" "49999010600" "49999010601"

© 2022 Rodriguez MI et al. *JAMA Network Open.*

		"49999010628""49999010630" "49999010660" "49999010690" "49999011600" "49999011630" "49999011660" "49999049530" "49999049560" "49999082030" "49999082060" "49999082090" "50268053111" "50268053115" "50268053211" "50268053213" "50742015401" "50742015405" "50742015410" "50742015490" "50742015501" "50742015505" "50742015510" "50742015590" "50742015601" "50742015605" "50742015610" "50742015690" "51079017201" "51079017220" "51079017301" "51079017308" "51079017401" "51079017420" "51079062620" "51079062720" "51079097201" "51079097217" "51079097219" "51079097220" "51079097230" "51079097256" "51079097257" "51079097301" "51079097320" "51079099501" "51079099520" "51138007430" "51138007530" "51138007630" "51138008330" "51138008430" "51138008530" "51138053710" "51138053730" "51138053810" "51138053830" "51138053910" "51138053930" "51138054010" "51138054030""51224000750" "51224000760" "51224010750" "51224010760" "52959020700" "52959020728" "52959020730" "52959020760" "52959086030" "52959086090" "52959089601" "52959089660" "53489046701" "53489046705" "53489046710" "53489046801" "53489046805" "53489046810" "53489046901" "53489046905" "53489046910" "53746017801" "53746017805" "53746017810" "53746017890" "53746017901" "53746017905" "53746021801" "53746021805" "53746021810" "53746021901" "53746021905" "53746021910" "53746022001" "53746022005" "53746022010" "54458097406" "54569420200" "54569420201" "54569420202" "54569420203" "54569474000" "54569474001" "54569478600" "54569478601" "54569525200" "54569525201" "54569525202" "54569535300" "54569535302" "54569535303" "54569535304" "54569535305" "54569535306" "54569536000" "54569536003" "54569536004" "54569536005" "54569536006" "54569537300""54569537302" "54569537303" "54569537304" "54569554600" "54569554601" "54569554602" "54569554603" "54868083000"

© 2022 Rodriguez MI et al. *JAMA Network Open.*

		"54868083001" "54868289400" "54868354500" "54868354501" "54868354502" "54868354503" "54868354600" "54868354601" "54868416000" "54868416001" "54868456100" "54868456101" "54868456102" "54868456103" "54868456104" "54868456400" "54868456401" "54868456402" "54868456403" "54868456404" "54868456405" "54868456600" "54868456601" "54868456602" "54868456603" "54868456604" "54868456900" "54868456901" "54868456902" "54868521700" "54868521701" "54868521702" "54868521703" "54868521704" "54868521705" "54868550500" "54868550501" "54868550502" "54868555800" "54868555801" "55045250401" "55045290400" "55045290402" "55045290406" "55045290500" "55045290508" "55045290600" "55045290601" "55045290602" "55045290606" "55045290608" "55045290609""55045304501" "55045304506" "55045304508" "55045376108" "55154358604" "55154358607" "55154358704" "55154358707" "55154358804" "55154358807" "55289021160" "55289038430" "55289038460" "55289038486" "55289038490" "55289038493" "55289038494" "55289061514" "55289061530" "55289061560" "55289061586" "55289061590" "55289061593" "55289061594" "55289061598" "55289091930" "55289091960" "55289091990" "55289091993" "55289091994" "55289091998" "55289093430" "55289093460" "55289093493" "55289093494" "55289093498" "55700004130" "55700024830" "55700024860" "55700024890" "55700032060" "55887041430" "55887041460" "55887041490" "55887057130" "55887057160" "55887057190" "55887061430" "55887061490" "55887062730" "55887062760" "55887062790" "55887062792" "55887094030" "55887094090" "57664039713" "57664039718" "57664039751" "57664039753" "57664039758""57664039788" "57664039799" "57664043513" "57664043518" "57664043551" "57664043553" "57664043558" "57664043588" "57664043599" "57664047413" "57664047418" "57664047451" "57664047453" "57664047458" "57664047488" "57664047499" "57866905401"

© 2022 Rodriguez MI et al. *JAMA Network Open.*

		"57866905402" "57866905403" "57866905404" "57866905405" "57866905501" "57866905502" "57866905503" "57866905504" "57866905505" "57866905601" "57866905602" "57866905603" "57866905604" "57866905605" "57866905606" "57866905901" "58016021300" "58016021310" "58016021330" "58016021360" "58016045700" "58016045710" "58016045730" "58016045760" "58016046600" "58016046630" "58016046660" "58016046690" "58016053600" "58016053630" "58016053660" "58016053690" "58016055300" "58016055310" "58016055330" "58016055360" "58016077200" "58016077230" "58016077260" "58016077290" "58016088300" "58016088330" "58016088360""58016088390" "58517004090" "58864001528" "58864001530" "58864001560" "58864001590" "58864050730" "58864069330" "58864069360" "58864078930" "58864078960" "58864085130" "59630057460" "59630057560" "59762432000" "59762432002" "59762432006" "59762432100" "59762432102" "59762432106" "59762432200" "59762432202" "59762432206" "60429011101" "60429011110" "60429011112" "60429011118" "60429011127" "60429011136" "60429011145" "60429011160" "60429011190" "60429011201" "60429011205" "60429011218" "60429011227" "60429011260" "60429011290" "60429011305" "60429011310" "60429011318" "60429011360" "60429011390" "60429028001" "60429028010" "60429028018" "60429072218" "60429072260" "60429072312" "60429072318" "60429072360" "60429072418" "60429072460" "60429072505" "60429072518" "60429072560" "60429072590" "60505019000" "60505019001" "60505019004""60505019008" "60505019100" "60505019101" "60505019104" "60505019108" "60505019200" "60505019201" "60505019204" "60505019208" "60505026001" "60505026002" "60505026007" "60505132901" "60687014301" "60687014311" "60687015501" "60687015511" "60687016201" "60687016211" "60760004260" "60760097360" "60760097398" "60760097460" "60760097490" "60760097560" "60760097590"

© 2022 Rodriguez MI et al. *JAMA Network Open.*

		"61073003005" "61073003105" "61073003205"<br>"61442036101" "61442036105" "61442036110"<br>"61442036201" "61442036205" "61442036301"<br>"61442036305" "61919031360" "61919039760"<br>"61919045060" "62022057460" "62022057560"<br>"62037057101" "62037057110" "62037057701"<br>"62037057710" "62037067401" "62037067405"<br>"62037067410" "62037067501" "62037067505"<br>"62037067510" "62037067601" "62037067605"<br>"62037067610" "62584018208" "62584025901"<br>"62584025911" "62584025980" "62584025985"<br>"62584033201""62584033211" "62584045201"<br>"62584045211" "62584045280" "62584045285"<br>"62756014201" "62756014202" "62756014301"<br>"63304020601" "63304020602" "63304076701"<br>"63304086001" "63304086005" "63304086030"<br>"63629139501" "63629139502" "63629139503"<br>"63629139601" "63629139602" "63629139701"<br>"63629139702" "63629139703" "63629139704"<br>"63629139705" "63629288301" "63629288302"<br>"63629288303" "63739029910" "63739030010"<br>"63739030110" "63739064010" "63739070210"<br>"63874050101" "63874050104" "63874050110"<br>"63874050114" "63874050120" "63874050124"<br>"63874050128" "63874050130" "63874050160"<br>"63874050190" "63874063501" "63874063510"<br>"63874063520" "63874063528" "63874063530"<br>"63874063560" "63874063590" "63874097401"<br>"63874097430" "63874097460" "64679052804"<br>"64679052805" "64679052904" "64679052905"<br>"64679053004" "64679053005" "65084041618"<br>"65084041620""65162017410" "65162017411"<br>"65162017450" "65162017510" "65162017511"<br>"65162017550" "65162017710" "65162017711"<br>"65162017750" "65162017910" "65162021810"<br>"65162021811" "65162021850" "65162021910"<br>"65162021911" "65162021950" "65162022010"<br>"65162022011" "65162022050" "65243023909"<br>"65243023918" "65243023927" "65243028806"<br>"65243028809" "65243028812" "65243028818"<br>"65243028906" "65243028909" "65243028912"<br>"65243028918" "65243037106" "65243037109"<br>"65243037206" "65243037209" "65243037218"

© 2022 Rodriguez MI et al. *JAMA Network Open.*

		"65243037309" "65862000801" "65862000805" "65862000890" "65862000899" "65862000901" "65862000905" "65862000990" "65862001001" "65862001005" "65862001046" "65862001090" "65862001099" "65862029101" "65862029105" "65862029201" "66116028260" "66116029330" "66116045430" "66116069560" "66267049360" "66336027030" "66336027060" "66336027090" "66336029260""66336035830" "66336035860" "66336035890" "66336088330" "66336088360" "66336088414" "66336088428" "66336088430" "66336088460" "66336088462" "66336088490" "66689001160" "67544004730" "67544004753" "67544004760" "67544004770" "67544004775" "67544004780" "67544004790" "67544004792" "67544004794" "67544004796" "67544010753" "67544010760" "67544010780" "67544010792" "67544016330" "67544016345" "67544016353" "67544016360" "67544016380" "67544042160" "67544042180" "67544042192" "67544042230" "67544042253" "67544042260" "67544042270" "67544042275" "67544042280" "67544042290" "67544042292" "67544042294" "67544042296" "67544042430" "67544042453" "67544042460" "67544042470" "67544042475" "67544042480" "67544042490" "67544042492" "67544042494" "67544042496" "67544042845" "67544042853" "67544042860" "67544042880" "67544048653" "67544059653""67544059660" "67544059680" "67544059696" "67544108180" "67544108194" "67544119853" "67544119860" "67544119880" "67544121653" "67544121660" "67544121680" "67544121692" "67544137980" "67544138360" "67544138380" "67767011501" "67877015901" "67877015905" "67877015910" "67877021701" "67877021705" "67877021710" "67877021801" "67877021805" "67877021810" "67877022101" "67877022105" "67877022110" "68012000213" "68012000316" "68030905601" "68084007201" "68084007211" "68084081932" "68084081933" "68115023030" "68115023060" "68115023130" "68115023160" "68115023190" "68115023230" "68115023245" "68115023260" "68180033607"

		"68180033707" "68180033801" "68180033909"<br>"68258101601" "68258107701" "68382002705"<br>"68382002801" "68382002805" "68382002810"<br>"68382002901" "68382002905" "68382002910"<br>"68382003001" "68382003005" "68382003010"<br>"68382003905""68382075801" "68382075805"<br>"68382075810" "68382075901" "68382075905"<br>"68382075910" "68382076001" "68382076005"<br>"68382076010" "68462015901" "68462015905"<br>"68462015910" "68462015918" "68462015990"<br>"68462016001" "68462016005" "68462016010"<br>"68462016018" "68462016090" "68462016101"<br>"68462016105" "68462016110" "68462016118"<br>"68462016190" "68645012059" "68645029059"<br>"68645030059" "00002210322" "00002210722"<br>"00364223201" "00364223301" "00405402401"<br>"00405402501" "00555044202" "00555044302"<br>"00603206121" "00839738606" "00839738706"<br>"00904199060" "00904199160" "00069393066"<br>"00069394041" "00069394066" "00069394071"<br>"00069394082" "00182185101" "00182185189"<br>"00182185201" "00182185210" "00247104530"<br>"00247104545" "00349892801" "00349892810"<br>"00349892901" "00349892905" "00364051001"<br>"00364051002" "00364069901" "00378019701"<br>"00378019705" "00378021001" "00378021010"<br>"00403005201" "00403005230" "00403005401"<br>"00403005430" "00403005471" "00405420501"<br>"00405420601" "00405420603" "00536346201"<br>"00536346205" "00536346501" "00536346505"<br>"00536346510" "00603283521" "00603283528"<br>"00603283621" "00603283628" "00603283632"<br>"00615254113" "00663393066" "00663393073"<br>"00663394066" "00663394071" "00663394082"<br>"00677097101" "00677097201" "00677097210"<br>"00781161301" "00781161305" "00781162301"<br>"00781162305" "00781162310" "00839701106"<br>"00839701112" "00839701116" "00839701206"<br>"00839701216" "00904022540" "00904022560"<br>"00904022561" "00904022660" "00904022661"<br>"00904022680" "49727010802" "49727010804"<br>"49727010902" "49727010905" "50111037201"<br>"50111037202" "50111037203" "50111037301"

		"50111037302" "50111037303" "50111037306" "51079020201" "51079020220" "51079020301" "51079020320" "52555007701" "52555007810" "54569020200" "54569020300" "54569020301" "54569020303" "54569020304" "54569201700" "54569201701" "54868003600" "54868003602" "54868003604" "54868087701" "55045121101" "55175385700" "55175385705" "55175385801" "55175385803" "55289006690" "55496150109" "55496150209" "57480031601" "57480031606" "57480031701" "57480031706" "58016090608" "58016090612" "58016090615" "58016090620" "58864010030" "60346063330" "60346063360" "60346063390" "60346078430" "00039022110" "00039022210" "00039022211" "00039022310" "00039022311" "00093725401" "00093725501" "00093725601" "00093725652" "00378401101" "00378401201" "00378401301" "00603374421" "00603374428" "00603374521" "00603374528" "00603374621" "00603374628" "00781504501" "00781504601" "00781504701" "10544021730" "10544021930" "10768715001" "10768747501" "10768770001" "16729000101" "16729000116" "16729000201" "16729000216" "16729000301" "16729000316" "21695074630" "21695074690" "21695074730" "21695074760" "21695074790" "21695099372" "23629002201" "23629002210" "35356089630" "42291051701" "42571010001" "42571010005" "42571010101" "42571010105" "42571010301" "42571010305" "43063003430" "43063003490" "43063012130" "43063012190" "43063012230" "43063012290" "43063058790" "43063069790" "43063069793" "43063069890" "43063069893" "43063069990" "43063069993" "45802077078" "45802082278" "45802094778" "49884084501" "49884084601" "49884084610" "49884084701" "49999078100" "49999078130" "49999078160" "49999078190" "49999080700" "49999080730" "49999080760" "49999080790" "51079042501" "51079042520" "51079042601" "51079042620" "51138033730" "51138033830" "51138033930" "52959088800" "52959088830" "52959093630" "52959093660" "54458096610"

© 2022 Rodriguez MI et al. *JAMA Network Open.*

		"54458096710" "54458096810" "54458097110"<br>"54458097210" "54458097310" "54569445300"<br>"54569445301" "54569470600" "54569585500"<br>"54569585501" "54569585502" "54569585503"<br>"54569607200" "54569607201" "54569607202"<br>"54569607203" "54868332700" "54868337700"<br>"54868337701" "54868337702" "54868420500"<br>"54868420501" "54868420502" "54868420600"<br>"54868420601" "54868420603" "54868420604"<br>"54868441200" "54868441201" "54868441202"<br>"54868545700" "54868545701" "54868545702"<br>"55111032001" "55111032005" "55111032101"<br>"55111032105" "55111032201" "55111032205"<br>"55700025630" "55700025660" "55700025690"<br>"55887022260" "55887022290" "57866707301"<br>"57866707302" "57866707401" "57866707402"<br>"58016000500" "58016000530" "58016000560"<br>"58016000590" "58016046700" "58016046730"<br>"58016046760" "58016046790" "58016084400"<br>"58016084430" "58016084460" "58016084490"<br>"60760022930" "60760023030" "61442011501"<br>"61442011505" "61442011601" "61442011605"<br>"61442011701" "61442011705" "63304042501"<br>"63304042601" "63304042701" "63629125501"<br>"63629125502" "63629304301" "63629304302"<br>"66993016202" "66993016302" "66993016402"<br>"67263019301" "68001017700" "68001017703"<br>"68001017800" "68001017803" "68001017900"<br>"68001017903""68084032601" "68084032611"<br>"68084032701" "68084032711" "68084078811"<br>"68084078821" "68084078825" "68084078895"<br>"68115084000" "69452012820" "69452012830"<br>"69452012920" "69452012930" "69452013020"<br>"69452013030" "69543012310" "69543012350"<br>"69543012410" "69543012450" "69543012510"<br>"69543012550" "76439012310" "76439012410"<br>"76439012510" "00047046324" "00047046330"<br>"00047046424" "00047046430" "00049017001"<br>"00049017402" "00049017403" "00049017807"<br>"00049017808" "00049155066" "00049155073"<br>"00049156066" "00049156073" "00049162030"<br>"00049411041" "00049411066" "00049411073"<br>"00049412041" "00049412066" "00049412073"

© 2022 Rodriguez MI et al. *JAMA Network Open.*

		"00172364900" "00172364910" "00172364960" "00172364970" "00172365000" "00172365010" "00172365060" "00172365070" "00182199400" "00182199401" "00182199405" "00182199489" "00182199500" "00182199501" "00182199505" "00182199589""00228289803" "00228289910" "00228289950" "00228289996" "00228290010" "00228290011" "00228290050" "00228290096" "00247044400" "00247044430" "00364260505" "00378034093" "00378034201" "00378034210" "00378043101" "00378043110" "00378110501" "00378110505" "00378111001" "00378111005" "00405538001" "00405538002" "00405538101" "00405538102" "00440756514" "00440756530" "00440756560" "00440756590" "00440756630" "00440756660" "00440756690" "00440756691" "00440756692" "00440756890" "00440756892" "00440756990" "00440756992" "00536569701" "00536569801" "00536570201" "00536570205" "00536570301" "00536570305" "00591046001" "00591046005" "00591046010" "00591046101" "00591046105" "00591046110" "00591084401" "00591084410" "00591084415" "00591084501" "00591084510" "00591084515" "00591090030" "00603375521" "00603375528" "00603375621" "00603375628""00615359529" "00615359543" "00615359553" "00615359563" "00615359565" "00615359629" "00615359643" "00615359653" "00615359663" "00615359665" "00662411041" "00662411066" "00662411073" "00662412041" "00662412066" "00662412073" "00677154401" "00677154405" "00677154501" "00677154505" "00781145201" "00781145210" "00781145213" "00781145301" "00781145310" "00781145313" "00832049110" "00832049111" "00832049210" "00832049211" "00839793906" "00839793912" "00839793916" "00839794006" "00839794012" "00839794016" "00904612361" "00904612461" "00904792440" "00904792460" "00904792461" "00904792480" "00904792540" "00904792560" "00904792561" "00904792580" "10370019001" "10370019005" "10370019101" "10370019105" "12280001200" "12280025930" "16729013900"

© 2022 Rodriguez MI et al. *JAMA Network Open.*

		"16729013916" "16729014000" "16729014016" "17236044101" "17236044105" "17236044110" "17236044201""17236044205" "17236044210" "21695046930" "21695046960" "21695046978" "21695046990" "21695047000" "21695047030" "21695047060" "21695047078" "21695047090" "21695096730" "23490563201" "23490563202" "23490563203" "23490563301" "23490563403" "23490563503" "23629013310" "33358015730" "33358015760" "33358015800" "33358015830" "33358015860" "35356009960" "35356012190" "35356087530" "35356089930" "35356089990" "35356093130" "35356093160" "35356093190" "43353036953" "43353036960" "43353036970" "43353036980" "43353036992" "43353036994" "43353037960" "43353037973" "43353037980" "43353037992" "43353037998" "49884045101" "49884045105" "49884045201" "49884045205" "49884074501" "49884074505" "49884074601" "49884074605" "49999010700" "49999010720" "49999010730" "49999010760" "49999010790" "49999010800" "49999010830" "49999010860" "49999010890""49999051430" "50111058401" "50111058402" "50111058501" "50111058502" "51079081001" "51079081017" "51079081019" "51079081020" "51079081023" "51079081024" "51079081101" "51079081117" "51079081119" "51079081120" "51138025130" "51138025230" "51138026530" "51138026630" "51138026730" "51138026830" "51138026930" "51138027030" "51138027130" "51285059802" "51285059804" "51285059805" "51285059902" "51285059904" "51285059905" "52189029124" "52189029129" "52189029224" "52189029229" "52544046001" "52544046005" "52544046010" "52544046101" "52544046105" "52544046110" "52959082200" "52959082230" "52959082260" "52959082320" "52959082360" "54569020601" "54569020602" "54569020603" "54569020700" "54569020701" "54569020702" "54569384100" "54569384101" "54569384102" "54569384103" "54569384200" "54569384201" "54569384202" "54569384204" "54569384205""54569384206" "54569393700"

		"54569393800" "54569393801" "54569554700" "54569554701" "54569554702" "54569554800" "54569554801" "54569554802" "54868099701" "54868099702" "54868099704" "54868108900" "54868108901" "54868108902" "54868108903" "54868331801" "54868331802" "54868331803" "54868331804" "54868331805" "54868331901" "54868331902" "54868331903" "54868331904" "54868331905" "54868331906" "54868331907" "54868333400" "54868333401" "54868333402" "54868333403" "54868333404" "54868333500" "54868333501" "54868333502" "54868333503" "54868442000" "54868498800" "54868498801" "54868498802" "54868498803" "54868498804" "54868521000" "54868521001" "54868521002" "54868521003" "54868536400" "54868536401" "54868536402" "55045205801" "55045226501" "55045226601" "55045330001" "55175385503" "55175385603" "55175385606" "55175517503" "55175517606""55289002790" "55289012530" "55289030190" "55289030193" "55289042430" "55289077907" "55289080614" "55289080630" "55289080660" "55289080686" "55289080690" "55289080693" "55289097601" "55289097614" "55289097630" "55289097660" "55289097690" "55289097693" "55700002230" "55887017930" "55887021230" "55887021260" "55887072790" "55887096830" "55887096930" "55953052401" "55953052440" "55953052470" "55953052480" "55953052501" "55953052540" "55953052570" "55953052580" "57480039101" "57480039106" "57480039201" "57480039206" "57664039813" "57664039818" "57664039888" "57664039913" "57664039918" "57664039988" "57866023601" "57866630201" "57866646201" "57866646202" "57866646301" "57866646302" "58016033400" "58016033402" "58016033430" "58016033460" "58016033490" "58016037600" "58016037602" "58016037630" "58016037660" "58016037690" "58016037699""58016069100" "58016069130" "58016069160" "58016069190" "58016087600" "58016087610" "58016087612" "58016087614" "58016087615" "58016087620" "58016087621"

		"58016087624" "58016087628" "58016087630" "58016087640" "58016087650" "58016087660" "58864002714" "58864002730" "58864002760" "58864002790" "58864016130" "58864016160" "58864068930" "58864068960" "58864070530" "58864085830" "58864095630" "58864095730" "59762054001" "59762054101" "59762054102" "59762054201" "59762054202" "59762503101" "59762503201" "59762503202" "59762503301" "59762503302" "60346045730" "60346061330" "60429008201" "60429008210" "60429008301" "60429008310" "60429008330" "60429008360" "60505014100" "60505014101" "60505014102" "60505014108" "60505014200" "60505014201" "60505014202" "60505014204" "60505014208" "60760002460" "60760014160" "60951071170" "60951071185""60951071470" "60951071485" "61392006330" "61392006331" "61392006332" "61392006339" "61392006345" "61392006351" "61392006354" "61392006360" "61392006390" "61392006391" "61392006430" "61392006431" "61392006432" "61392006439" "61392006445" "61392006451" "61392006454" "61392006460" "61392006490" "61392006491" "61919028690" "62037087130" "62037087201" "62037087205" "62037087301" "62037087305" "62269029124" "62269029129" "62269029224" "62269029229" "62584071601" "62584071633" "62584071733" "63629139401" "63629139402" "63629139403" "63629139801" "63629139802" "63629139803" "63739011601" "63739011602" "63739011603" "63739011610" "63739011615" "63739011701" "63739011702" "63739011703" "63739011710" "63739011715" "63874031601" "63874031604" "63874031605" "63874031610" "63874031612" "63874031614" "63874031615" "63874031620" "63874031621""63874031624" "63874031628" "63874031630" "63874031650" "63874031660" "63874031681" "63874031690" "63874043201" "63874043204" "63874043210" "63874043214" "63874043220" "63874043221" "63874043224" "63874043228" "63874043230" "63874043260" "63874043281" "63874043290" "65243018318"

© 2022 Rodriguez MI et al. *JAMA Network Open.*

		"65243034609" "65243037809" "66267010030" "66267010060" "66336026930" "66336066230" "66336066260" "66336066290" "66336073030" "66336073060" "66336073090" "67544009753" "67544009760" "67544009770" "67544009780" "67544009792" "67544009794" "67544012953" "67544012960" "67544012970" "67544012980" "67544012994" "67544019980" "67544030232" "67544030245" "67544030260" "67544030273" "67544030280" "67544030292" "67544030298" "67544075160" "67544075180" "67544075660" "67544075680" "67544080860" "68030646201" "68084011101" "68084011111" "68084011201" "68084011211""68084029511" "68084029521" "68115015430" "68115015460" "68115015530" "68115015560" "68115015590" "68115061600" "68258101201" "68258101301" "68258105101" "68645015054" "68645015159" "71114022100" "71114022101" "71114022105" "71114022200" "71114022201" "71114022205" "00009013101" "00009014101" "00009014102" "00009014106" "00009017103" "00009017105" "00009017106" "00009017107" "00009017111" "00009017112" "00009017113" "00039005110" "00039005111" "00039005150" "00039005210" "00039005211" "00039005250" "00039005270" "00039005305" "00093834201" "00093834301" "00093834305" "00093834310" "00093834398" "00093834401" "00093834405" "00093834410" "00093834419" "00093834493" "00093834498" "00093936401" "00093936405" "00093936410" "00093943301" "00093943305" "00093947753" "00182121919" "00182122001" "00182122005" "00182122101" "00182122105" "00182122110" "00182264501" "00182264600" "00182264601" "00182264605" "00182264689" "00182264700" "00182264701" "00182264705" "00182264710" "00182264789" "00247127000" "00247127006" "00364257701" "00364257705" "00364257801" "00405536050" "00405536101" "00405536102" "00405536201" "00405536203" "00405537601" "00405537603" "00440757020" "00440757114" "00440757130" "00440757160" "00440757190" "00440757191"

© 2022 Rodriguez MI et al. *JAMA Network Open.*

		"00440757192" "00440757194" "00440757195" "00536564201" "00536564301" "00536564305" "00536575101" "00536575201" "00536575205" "00603376221" "00603376321" "00615155629" "00615155643" "00615155653" "00615155663" "00615450929" "00615450943" "00615450953" "00615450963" "00677158001" "00677158101" "00677158105" "00781113801" "00781114601" "00781119101" "00781119110" "00781145501" "00781145601" "00781145613" "00781145701" "00781145705""00781145710" "00781145713" "00839803906" "00839804006" "00839804016" "00839804106" "00839804116" "00904507560" "00904507660" "00904507740" "00904507760" "00904507780" "00904613760" "00904613840" "00904613860" "00904613960" "00904613980" "21695046730" "21695046760" "21695046830" "21695046860" "21695046872" "21695046878" "23155005601" "23155005701" "23155005801" "23155005810" "23490563801" "23490563901" "23490563902" "33358016030" "33358016060" "33358016101" "33358016130" "33358016160" "35356036030" "35356036060" "35356036090" "35356099530" "35356099560" "35356099590" "38245036410" "38245036420" "38245036450" "38245036455" "38245043310" "38245043350" "38245043355" "38245047749" "42254009030" "42254009060" "42254009090" "42291031650" "42291031710" "43063043314" "43063043330" "43063043386" "43063043390" "43063043393" "43353058280""43353065653" "43353065660" "43353065670" "43353065680" "43353065690" "43353065692" "43353065694" "43353065960" "43353065980" "44514038536" "49999011300" "49999011301" "49999011330" "49999011360" "49999011390" "51079087201" "51079087217" "51079087219" "51079087220" "51079087301" "51079087317" "51079087319" "51079087320" "51138037030" "51138037130" "51138037210" "51138037230" "51655075324" "51655075352" "51655075877" "51655090424" "51655094724" "51655094725" "52817012010" "52817012110" "52817012200" "52817012210" "52959017730"

© 2022 Rodriguez MI et al. *JAMA Network Open.*

		"52959044901" "52959044930" "52959044960" "52959059890" "54569020000" "54569020002" "54569020003" "54569020400" "54569383000" "54569383001" "54569383002" "54569383100" "54569383101" "54569383102" "54569383104" "54569383108" "54569383109" "54569383200" "54868037301" "54868037302" "54868099601" "54868099602""54868124400" "54868124401" "54868124402" "54868124403" "54868124404" "54868124501" "54868124502" "54868124503" "54868124505" "54868168801" "54868326500" "54868326501" "54868326503" "54868326504" "54868326505" "54868326601" "54868326602" "54868326603" "54868326604" "54868342600" "54868342601" "55045213800" "55045213801" "55175523001" "55289017330" "55289060630" "55289060690" "55289061401" "55289061460" "55289089201" "55289089214" "55289089215" "55289089230" "55289089260" "55289089286" "55289089290" "55289089293" "55289089298" "55887053630" "55953034240" "55953034340" "55953034370" "55953034380" "55953034440" "55953034470" "55953034480" "57237002001" "57237002101" "57237002105" "57237002199" "57237002201" "57237002205" "57237002299" "57866023701" "57866640901" "57866640902" "57866640903" "57866640904" "57866640905" "57866640906""58016037800" "58016037802" "58016037830" "58016037860" "58016037890" "58016037899" "58864021430" "58864021460" "58864022430" "58864022460" "58864022493" "59762372501" "59762372603" "59762372703" "59762372704" "59762372706" "59762372707" "59762702009" "59762702105" "59762702109" "59762702205" "59762702209" "59930159201" "59930162201" "59930163901" "59930163902" "59930163903" "60346066230" "60346073030" "60346073060" "60346089030" "60346093830" "60429008512" "60429008518" "60429008527" "60429008530" "60429008536" "60429008560" "61392011730" "61392011731" "61392011732" "61392011739" "61392011745" "61392011751" "61392011754" "61392011760" "61392011790"

© 2022 Rodriguez MI et al. *JAMA Network Open.*

		"61392011791" "61392011930" "61392011931" "61392011932" "61392011939" "61392011945" "61392011951" "61392011954" "61392011960" "61392011990" "61392011991" "61392012030" "61392012031" "61392012032" "61392012039" "61392012045" "61392012051" "61392012054" "61392012060" "61392012090" "61392012091" "61919033030" "61919033060" "61919037860" "62584071801" "62584071833" "62584071901" "62584071933" "63629139301" "63629139302" "63629139303" "63629139304" "63629290701" "63629290702" "63739011801" "63739011802" "63739011803" "63739011810" "63739011815" "63739011901" "63739011902" "63739011903" "63739011910" "63739011915" "63874031701" "63874031704" "63874031710" "63874031712" "63874031714" "63874031715" "63874031720" "63874031724" "63874031728" "63874031730" "63874031740" "63874031750" "63874031760" "63874031790" "63874058801" "63874058804" "63874058810" "63874058814" "63874058820" "63874058830" "63874058860" "63874058880" "63874058890" "63874066501" "63874066504" "63874066510" "63874066514" "63874066530" "63874066560""63874066590" "64720012310" "64720012410" "64720012510" "64720012511" "64720029010" "64720029110" "64720029210" "64720029211" "65243017609" "65243017612" "65243017618" "65243017627" "65243017636" "65243018509" "65243018512" "65243018518" "65243018536" "65243028509" "65243028518" "65243032509" "65243032518" "65243034309" "65243034336" "65243037509" "65862002801" "65862002901" "65862002905" "65862003001" "65862003099" "66116044030" "66267010330" "66336002830" "66336093830" "66336093860" "67544009853" "67544009860" "67544009870" "67544009880" "67544009890" "67544009892" "67544009894" "67544047882" "67544054882" "67544056653" "67544056660" "67544056670" "67544056680" "67544056692" "67544056694" "67544061353" "67544061360" "67544061370" "67544061380" "67544061390" "67544061392"

© 2022 Rodriguez MI et al. *JAMA Network Open.*

		"67544061394" "67544064380" "67544064394" "67544065353""67544065360" "67544065370" "67544065380" "67544065390" "67544065392" "67544065394" "67544065398" "67544066141" "67544066181" "67544087560" "67544087580" "68084080701" "68084080711" "68115016030" "68115016130" "68115016160" "68258101401" "68382065601" "68382065701" "68382065705" "68382065801" "68382065810" "68645021054" "68645021154" "00009034101" "00009034102" "00009035201" "00009035202" "00009035203" "00009035204" "00009344901" "00009344903" "00093803401" "00093803501" "00093803505" "00093803510" "00093803601" "00143991801" "00143991901" "00143991905" "00143992001" "00143992005" "00143992010" "00378111301" "00378112501" "00378112510" "00378114201" "23629002301" "23629002310" "38245038110" "38245038120" "38245038150" "38245072510" "42254028130" "42254028190" "43063011990" "43063012090" "49999057160" "49999080800" "51138036730""51138036830" "51138036930" "52152013302" "52152013402" "52152013404" "52152013405" "52152013502" "52152013504" "52152013505" "52544055801" "52544055805" "52544055810" "52544055901" "52544055905" "52544055910" "52544056001" "52544056005" "52544056010" "54569369000" "54569369001" "54569469500" "54569469501" "54868301700" "54868371100" "54868371101" "54868409100" "54868409101" "54868409102" "54868409103" "54868484200" "54868571200" "55370014607" "55370014608" "55370014609" "55370014707" "55370014708" "55370014709" "55370050607" "55370050608" "55370050609" "55953003440" "55953003441" "55953003540" "55953003541" "55953003570" "55953003580" "55953003640" "59762378101" "59762378201" "59762378203" "59762378301" "59762378302" "59762378303" "62269029324" "62269029424" "62269029429" "64909010107" "64909010207" "64909010208" "64909010407""64909010507" "64909010508" "66336071290" "67253046010" "67253046011"

© 2022 Rodriguez MI et al. *JAMA Network Open.*

		"67253046050" "67253046110" "67253046111" "67253046150" "67253046210" "67253046211" "67253046250" "68115062200" "00009007002" "00009011402" "00009011404" "00009011405" "00009047706" "00172297860" "00172297960" "00172297961" "00172297970" "00172297980" "00172298060" "00172298070" "00182164504" "00182164510" "00182167701" "00182167901" "00364072001" "00364072090" "00364072101" "00364072201" "00364072290" "00378021701" "00378055101" "00405502401" "00405502501" "00405502601" "00440856390" "00536473801" "00536473802" "00536473901" "00536473902" "00536473905" "00536474401" "00603609621" "00603609721" "00603609723" "00603609732" "00603609821" "00603609828" "00677095301" "00677095401" "00677095404" "00677095501" "00781192201" "00781193201" "00781194213" "00839675506""00839675516" "00839701406" "00839701606" "00904023460" "00904023525" "00904023640" "00904023660" "51079029120" "51079029220" "51079029320" "52555029101" "52555029201" "52555029301" "53489015201" "53489015210" "53489015301" "53746028601" "53746028602" "53746028610" "53746028701" "53746028705" "54569020502" "54569020800" "54569020801" "54569167800" "54868102000" "55175385901" "55175386001" "55289026590" "56126010311" "58016037030" "68084055611" "68084055621" "00009010002" "00009010003" "00009010005" "00009010011" "00172224560" "00172224570" "00172224580" "00182108401" "00182108405" "00364047701" "00364047702" "00378021501" "00378021505" "00405503101" "00405503103" "00536466801" "00536466805" "00536466810" "00603612121" "00615151413" "00677059201" "00677059205" "00686224510" "00686224512" "00686406213" "00839625306" "00839625312" "00839625316" "00904022360" "00904022361" "00904022380" "49727031705" "51079056001" "51079056020" "53445108400" "54569021401" "54868136101"

© 2022 Rodriguez MI et al. *JAMA Network Open.*

Contraception - Sterilization	ICD-9/ICD-10/CPT	0U574ZZ, 0U578ZZ, 0UL74CZ, 0UL74DZ, 0UL74ZZ, 0UL78DZ, 0UL78ZZ, 58340, 58565, 58600, 58605, 58611, 58615, 58670, 58671, 59510, 59618, 662, 6621, 6622, 6629, 74740, A4264, V252, V2651, Z302, Z9851
Contraception-Implant	ICD-9/CPT	9963, 11981, A4260, J7306, J7307, S0180, V255, V4552
Contraception-IUD	ICD-9/ICD-10/CPT	0UH97HZ, 0UH98HZ, 0UHC7HZ, 0UHC8HZ, 58300, 697, 99665, J7297, J7298, J7300, J7301, J7306, S4989, Q0090, S4981, T8331XA, T8332XA, T8339XA, T8359XA, T836XXA, V2511, V4551, Z975, Z30014, Z30430, Z30431
Contraception-Oral Contraception	NDC	00008005601, 00008005602, 00008006201, 00008006202, 00008007501, 00008007502, 00008007801, 00008007802, 00008091202, 00008111720, 00008111730, 00008251101, 00008251102, 00008251401, 00008251402, 00008251403, 00008253301, 00008253302, 00008253303, 00008253501, 00008253505, 00008253601, 00008253603, 00008253605, 00008257601, 00008257602, 00009074630, 00009074631, 00009074634, 00009074635, 00009348404, 00009348405, 00009348406, 00009348410, 00009470901, 00009470913, 00009737601, 00009737602, 00009737603, 00009737604, 00009737607, 00009737611, 00023586228, 00023586230, 00025007107, 00025007124, 00025008109, 00025008124, 00025008184, 00025015107, 00025015124, 00025016109, 00025016124, 00025016184, 00025025203, 00025025403, 00025025706, 00025025906, 00025025912, 00025026306, 00025026506, 00025027206, 00025027212, 00025027406, 00025027412, 00047092635, 00047092735, 00047093011, 00047094211, 00047094435, 00047094735, 00052026106, 00052026108, 00052026906, 00052028106, 00052028306, 00052028308, 00062125100, 00062125101, 00062125115, 00062125120, 00062133115, 00062133215, 00062133220, 00062141101, 00062141116, 00062141123,

		00062171215, 00062171400, 00062171415, 00062171420, 00062176015, 00062176100, 00062176115, 00062176120, 00062177015, 00062177115, 00062178015, 00062178022, 00062178100, 00062178115, 00062178120, 00062178122, 00062179515, 00062179600, 00062179615, 00062190015, 00062190115, 00062190120, 00062190215, 00062190315, 00062190320, 00062190700, 00062190715, 00062191000, 00062191015, 00062192001, 00062192015, 00071091315, 00071091345, 00071091347, 00071091348, 00071091547, 00071091548, 00071091647, 00071091648, 00071091715, 00071091745, 00071091747, 00071091748, 00071092815, 00071092847, 00087057841, 00087057941, 00087058342, 00093214028, 00093214062, 00093313482, 00093313491, 00093330416, 00093330428, 00093330516, 00093330528, 00093531528, 00093531581, 00093531628, 00093531681, 00093532828, 00093532862, 00093542328, 00093542358, 00093542362, 00093566128, 00093566158, 00093603182, 00093603191, 00093614882, 00093614891, 00093807316, 00093807328, 00247052028, 00247059101, 00247069028, 00247069128, 00247069228, 00247100328, 00247100421, 00247139828, 00247151328, 00247151628, 00247151728, 00247176404, 00247176421, 00247176521, 00247198621, 00247198628, 00247200828, 00247201004, 00247201008, 00247201028, 00247201228, 00247201328, 00247214728, 00247216928, 00247217028, 00247223028, 00247223528, 00247226028, 00247226828, 00339651899, 00378334016, 00378334053, 00378655053, 00378655085, 00378727253, 00378727285, 00378727753, 00378728053, 00378728153, 378728253, 00378728285, 00378728353, 00378728485, 00378728490, 00378728590, 00378728753, 00378729253, 00378729653, 00378729753, 00378729785, 00378729853, 00378730053, 00378730153, 00378730185, 00378730653, 00378730685,

© 2022 Rodriguez MI et al. *JAMA Network Open.*

		00378730753, 00378730785, 00378730853, 00378730885, 00378731553, 00378731585, 00378731685, 00430000531, 00430001005, 00430042014, 00430048214, 00430053014, 00430053514, 00430053550, 00430053595, 00430053714, 00430053750, 00430054014, 00430054050, 00430057014, 00430057045, 00430058014, 00430058045, 00430058114, 00430058214, 00430058311, 00430058514, 00430058545, 00536405548, 00536405648, 00536405748, 00536405844, 00536405944, 00555034458 00555071558, 00555900858, 00555900867, 00555900879, 00555900942, 00555900957, 00555900980, 00555901058, 00555901079, 00555901258, 00555901279, 00555901458, 00555901467, 00555901479, 00555901658, 00555901858, 00555902058, 00555902079, 00555902542, 00555902557, 00555902658, 00555902742, 00555902757, 00555902858, 00555903270, 00555903458, 00555903479, 00555904358, 00555904379, 00555904558, 00555904579, 00555904758, 00555904958, 00555904979, 00555905058, 00555905079, 00555905158, 00555905167, 00555905179, 00555906458, 00555906467, 00555906479, 00555906558, 00555906658, 00555906667, 00555906679, 00555912366, 00555913167, 00555913179, 00603359001, 00603359017, 00603359049, 00603751201, 00603751217, 00603751249, 00603752101, 00603752117, 00603752149, 00603752501, 00603752517, 00603752549, 00603754001, 00603754017, 00603754049, 00603760602, 00603760615, 00603760648, 00603760702, 00603760715, 00603760748, 00603760801, 00603760817, 00603760901, 00603760917, 00603761001, 00603761017, 00603761049, 00603762501, 00603762517, 00603762549, 00603763401, 00603763417, 00603763449, 00603764017, 00603764201, 00603764217, 00603766301, 00603766317, 00603766517, 00703680101, 00703680104, 00703681121, 00781405815, 00781406015, 00781406215,

© 2022 Rodriguez MI et al. *JAMA Network Open.*

		00781406615, 00781407515, 00781407552, 00781410315, 00781410352, 00781557515, 00781558307, 00781558315, 00781558336, 00781558436, 00781558491, 00781565615, 00781565815, 00905027928, 00905029128, 16714007301, 16714007304, 16714034001, 16714034004, 16714034601, 16714034604, 16714034701, 16714034704, 16714034801, 16714034804, 16714035901, 16714035903, 16714035904, 16714036001, 16714036004, 16714036301, 16714036304, 16714036501, 16714036504, 16714036603, 16714036701, 16714036704, 16714037001, 16714037003, 16714040401, 16714040404, 16714040501, 16714040504, 16714040601, 16714040604, 16714040701, 16714040703, 16714040801, 16714040803, 16714041301, 16714041304, 16714041601, 16714041603, 16714044001, 16714044004, 16714044101, 16714044104, 16714046401, 16714046404, 17314423101, 17314423102, 17478026006, 17478026028, 17478026106, 17478026128, 21695028128, 21695040701, 21695040703, 21695051428, 21695068528, 21695076901, 21695076928, 21695077001, 21695077028, 21695085501, 21695085601, 21695085603, 21695085701, 21695085703, 21695099528, 23490765301, 23490767001, 23490769901, 24090080184, 24090096184, 35356001468, 35356001568, 35356002168, 35356025528, 35356036128, 35356036328, 35356036528, 35356036828, 35356037028, 35356041128, 35356047605, 35356047628, 42254024228, 42254026028, 42254027028, 42254028703, 42254028728, 42987010023, 42987010124, 42987010719, 42987010813, 42987010923, 42987011014, 42987011061, 42987011124, 42987011128, 42987011423, 42987011427, 42987011524, 42987011528, 50102010000, 50102010001, 50102010003, 50102010010, 50102010048, 50102012001, 50102012003, 50102012010, 50102012048, 50102012801, 50102012803, 50102013001, 50102013003, 50102013010,

© 2022 Rodriguez MI et al. *JAMA Network Open.*

		50102013048, 50102013090, 50102015401, 50102015403, 50102023311, 50102023313, 50102023511, 50102023513, 50419040201, 50419040203, 50419040301, 50419040303, 50419040501, 50419040503, 50419040603, 50419040701, 50419040703, 50419040803, 50419040872, 50419040901, 50419040903, 50419041021, 50419041112, 50419041128, 50419042408, 50419043203 50419043206, 50419043303, 50419043306, 50419043312, 50452025115, 50458017100, 50458017115, 50458017600, 50458017615, 50458017800, 50458017815, 50458017820, 50458019112, 50458019115, 50458019120, 50458019400, 50458019406, 50458019411, 50458019412, 50458019416, 50458019423, 50458019428, 50458019601, 50458019615, 50458019712, 50458019715, 50458025100, 50458025106, 50458025115, 50458025128, 51285001728, 51285005866, 51285007997, 51285008070, 51285008198, 51285008297, 51285008370, 51285008498, 51285008787, 51285009158, 51285009287, 51285011458, 51285012058, 51285012570, 51285012698, 51285012797, 51285012870, 51285012998, 51285013197, 51285043165, 51285043187, 51285051428, 51285054628, 51285057628, 51660012786, 51660057286, 51862000701, 51862000706, 51862001201, 51862001206, 51862002801, 51862002806, 51862003601, 51862003603, 51862004501, 51862004591, 51862004701, 51862004791, 51862007201, 51862007206, 51862009701, 51862009706, 51862010001, 51862010006, 51862010201, 51862010206, 51862023801, 51862023803, 51862026001, 51862026006, 51862027901, 51862027906, 51862028401, 51862028403, 51862029201, 51862029206, 51862031801, 51862031803, 51862047001, 51862047006, 51862047101, 51862047106, 51862048965, 51862051001, 51862051006, 51862054501, 51862054506, 51862056401, 51862056406, 52544005431, 52544005841, 52544005872, 52544006431,

© 2022 Rodriguez MI et al. *JAMA Network Open.*

		52544008728, 52544008741, 52544014328, 52544014331, 52544016528, 52544016541, 52544016731, 52544016741, 52544017572, 52544020431, 52544021028, 52544021928, 52544022829, 52544022891, 52544023328, 52544023341, 52544023528, 52544023531, 52544024531, 52544024728, 52544024828, 52544024928, 52544024941, 52544025428, 52544025928, 52544025931, 52544025988, 525440
Contraception-Patch	NDC	62192024, 50458019201, 50458019215, 50458019224, 54569541300, 54868467000
Contraception-Ring	NDC	00052027301, 00052027303, 35356041003, 54569586500, 54868483200, 54868483201

© 2022 Rodriguez MI et al. *JAMA Network Open.*

eTable 3. Study Outcomes Among Emergency Medicaid Latina Recipients With Preexisting or Gestational Diabetes Following Prenatal Care Expansion from 2010-2019, N = 2907

	Treatment (Oregon) (N = 1834)			Comparison (South Carolina) (N = 1073)			Adjusted Difference-in-Difference Estimate
	Pre-policy (n = 694)	Post-policy (n = 1140)	Difference	Pre-policy (n = 454)	Post-policy (n = 619)	Difference	
	%	%	%	%	%	%	% (95% CI)
Any medication initiation[abd]	0.29	32.7	32.41	0.66	0.64	-0.02	31.8 (27.9, 35.7)
Insulin initiation[abe]	0	14.3	14.3	0.66	0	-0.66	14.4 (9.7, 19.1)
Gestational hypertension[bcf]	7.6	8.5	0.9	8.1	11.0	2.9	-2.3 (-6.8, 2.2)
Cesarean birth[bcg]	36.6	37.8	1.2	39.0	38.1	-0.9	3.3 (-4.5, 11.1)
Postpartum contraception (all)[bh]	0.14	24.7	24.56	4.0	4.4	0.4	22.9 (17.6, 28.2)
Postpartum sterilization[bi]	0	16.1	16.1	3.1	1.6	-1.5	17.5 (12.6, 22.4)
Infant composite morbidity[bj]	30.4	28.9	-1.5	30.8	36.3	5.5	-5.4 (-12.5, 1.7)

[a]At any point during pregnancy
[b]Estimates adjusted for maternal age, bmi
[c]Estimates adjusted for nulliparity
[d]pre-test of parallel trends, p = 0.98
[e]pre-test of parallel trends, p = 0.43
[f]pre-test of parallel trends, p = 0.65
[g]pre-test of parallel trends, p = 0.67
[h]pre-test of parallel trends, p = 0.86
[i]pre-test of parallel trends, p = 0.47
[j]pre-test of parallel trends, p = 0.52

© 2022 Rodriguez MI et al. *JAMA Network Open.*

eFigure 1. Cohort Creation

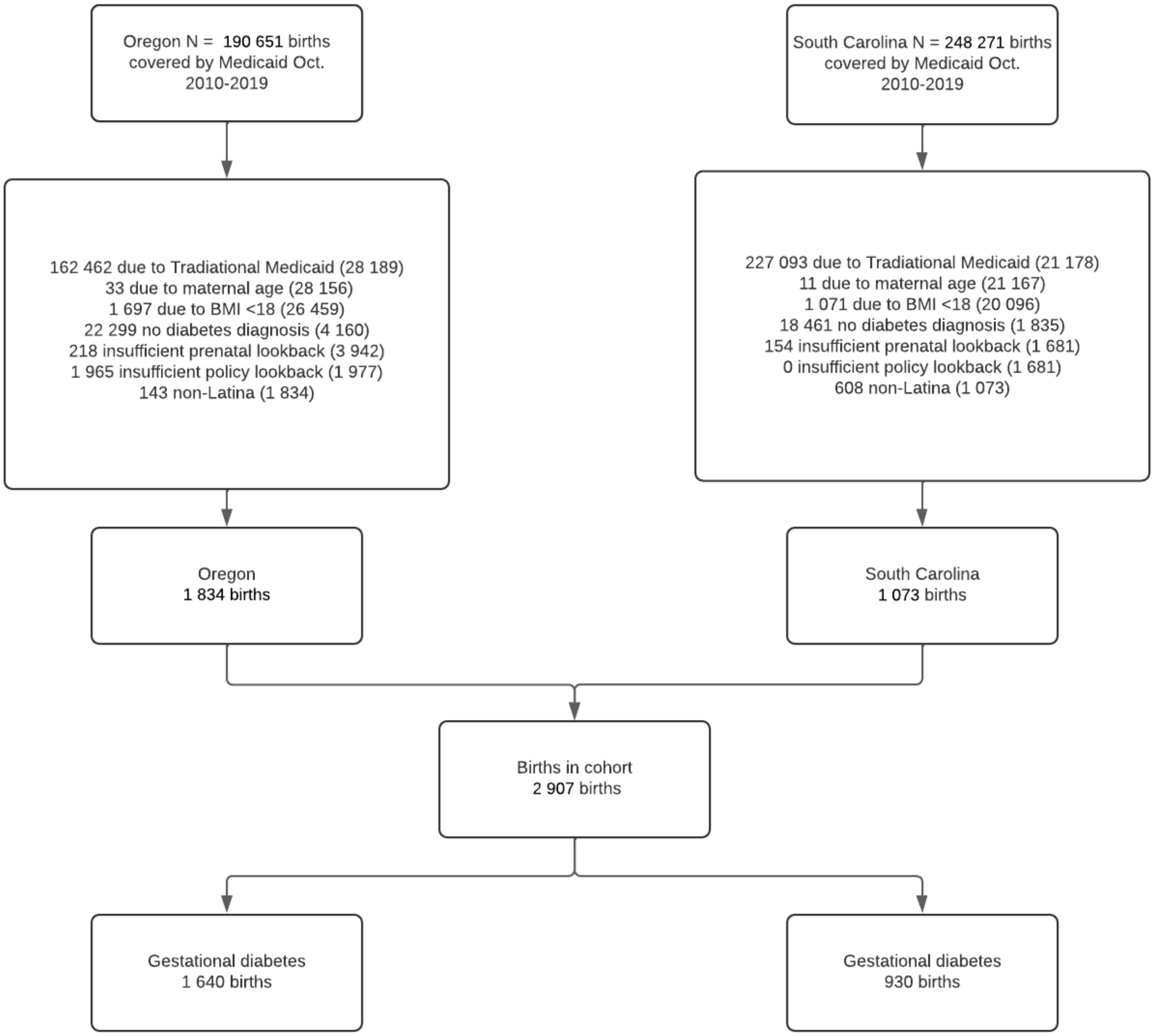

© 2022 Rodriguez MI et al. *JAMA Network Open.*

eFigure 2. Adjusted Trend Estimates of any Medication Initiation During Prenatal Period Among Emergency Medicaid Recipients With Pregestational or Gestational Diabetes (2010-2019)

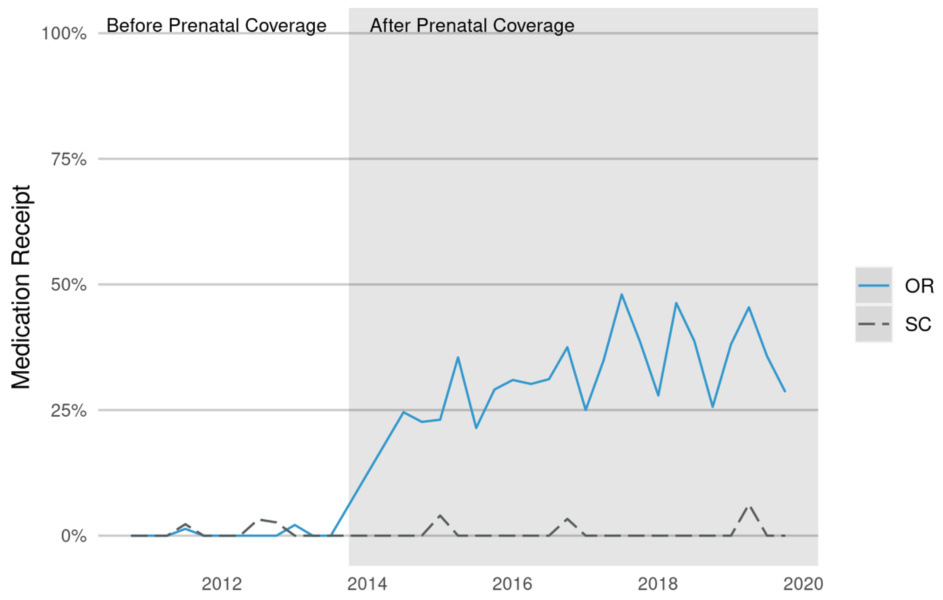

© 2022 Rodriguez MI et al. *JAMA Network Open.*

eFigure 3. Adjusted Trend Estimates of Insulin Initiation During Prenatal Period Among Emergency Medicaid Recipients With Pregestational or Gestational Diabetes (2010-2019)

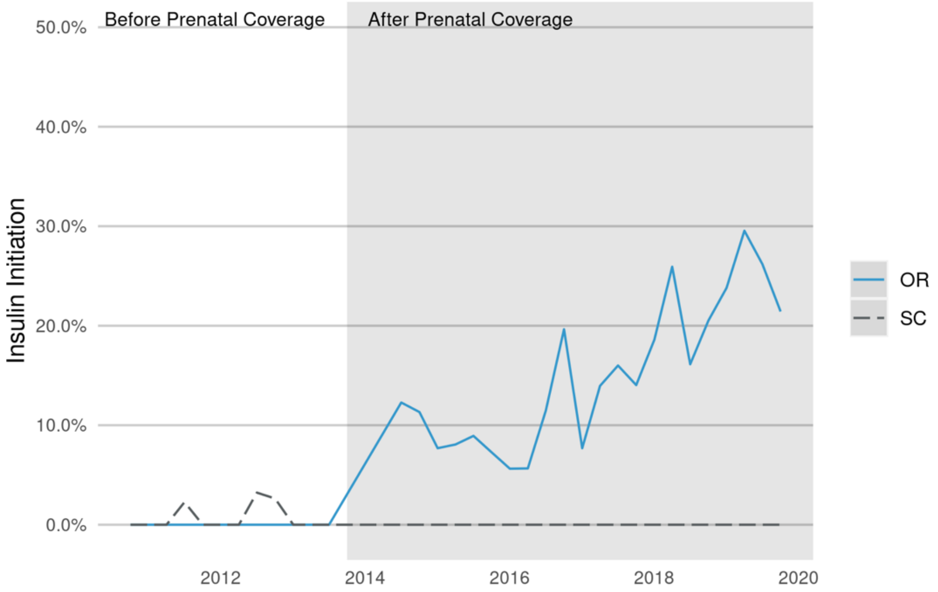

© 2022 Rodriguez MI et al. *JAMA Network Open.*

eFigure 4. Adjusted Trend Estimates of Gestational Hypertension During Prenatal Period Among Emergency Medicaid Recipients With Pregestational or Gestational Diabetes (2010-2019)

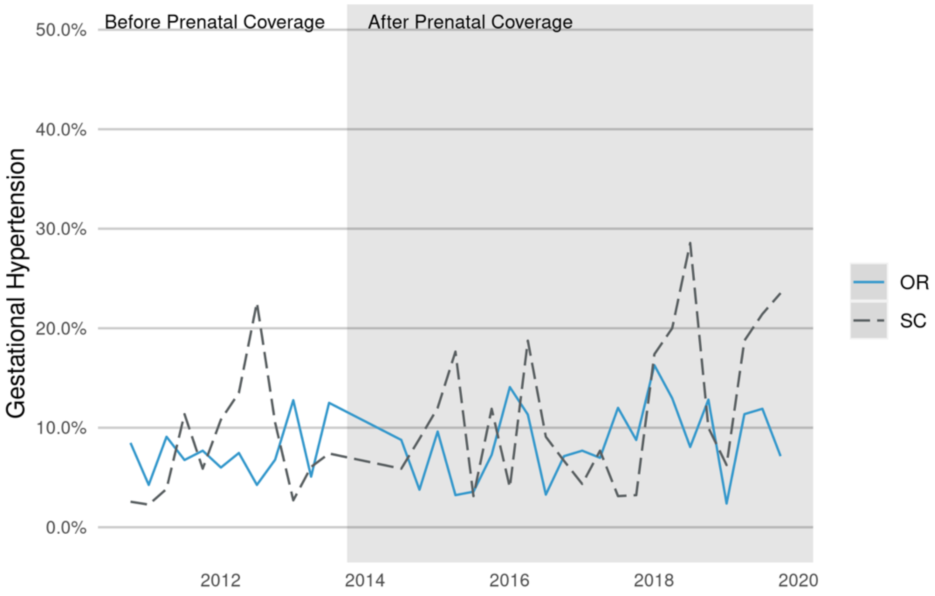

© 2022 Rodriguez MI et al. *JAMA Network Open.*

eFigure 5. Adjusted Trend Estimates of Cesarean Birth During Prenatal Period Among Emergency Medicaid Recipients With Pregestational or Gestational Diabetes (2010-2019)

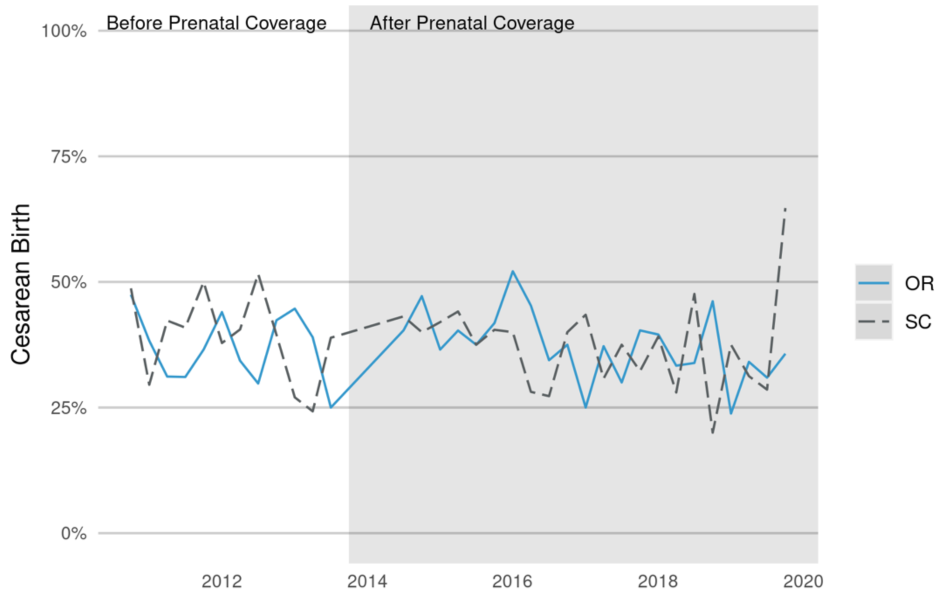

© 2022 Rodriguez MI et al. *JAMA Network Open.*

eFigure 6. Adjusted Trend Estimates of Postpartum Contraception During Prenatal Period Among Emergency Medicaid Recipients With Pregestational or Gestational Diabetes (2010-2019)

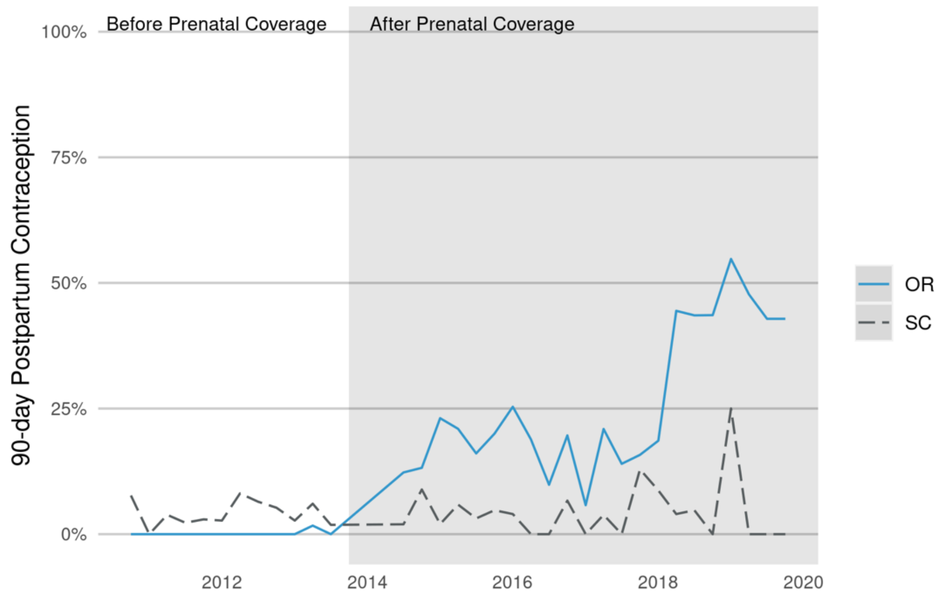

eFigure 7. Adjusted Trend Estimates of Postpartum Sterilization During Prenatal Period Among Emergency Medicaid Recipients With Pregestational or Gestational Diabetes (2010-2019)

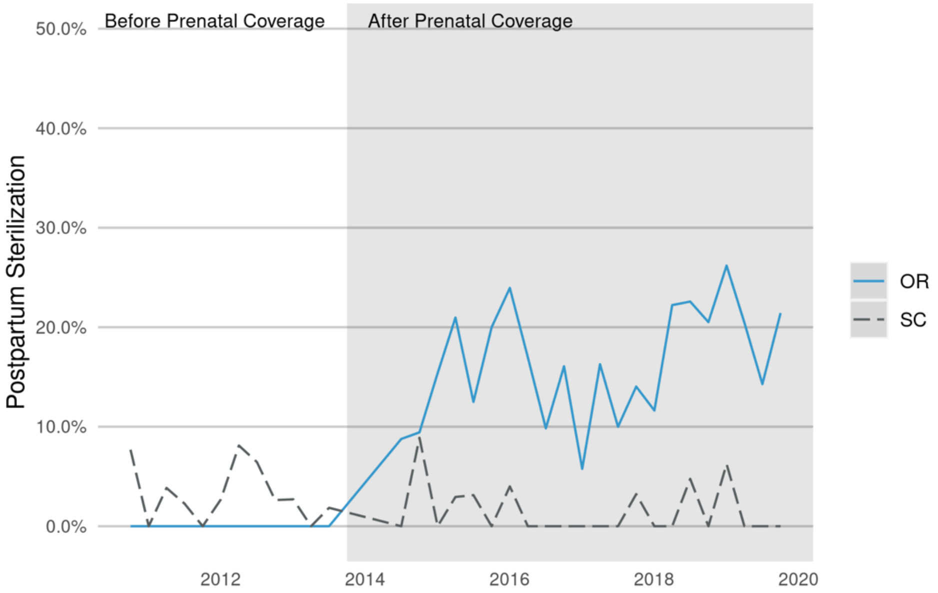

© 2022 Rodriguez MI et al. *JAMA Network Open.*

eFigure 8. Adjusted Trend Estimates of Infant Morbidity During Prenatal Period Among Emergency Medicaid Recipients With Pregestational or Gestational Diabetes (2010-2019)

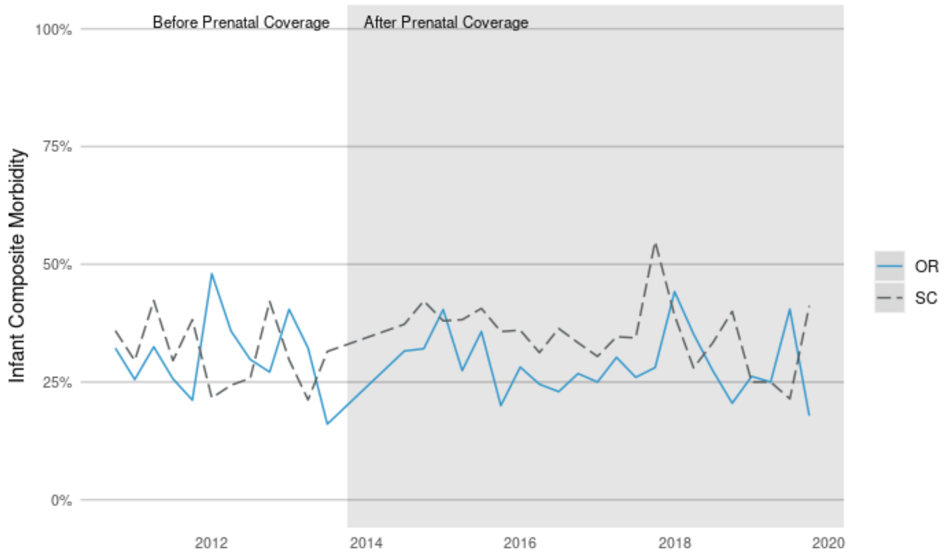

© 2022 Rodriguez MI et al. *JAMA Network Open.*
